# Integrated ‘Omics’, Targeted Metabolite and Single-cell Analyses of Arctic Snow Algae Functionality and Adaptability

**DOI:** 10.3389/fmicb.2015.01323

**Published:** 2015-11-25

**Authors:** Stefanie Lutz, Alexandre M. Anesio, Katie Field, Liane G. Benning

**Affiliations:** ^1^Cohen Laboratories, School of Earth and Environment, University of LeedsLeeds, UK; ^2^GFZ German Research Centre for GeosciencesPotsdam, Germany; ^3^Bristol Glaciology Centre, School of Geographical Sciences, University of BristolBristol, UK; ^4^Department of Animal and Plant Sciences, University of SheffieldSheffield, UK

**Keywords:** snow algae, Svalbard, metabolomics, genomics, single-cell

## Abstract

Snow algae are poly-extremophilic microalgae and important primary colonizers and producers on glaciers and snow fields. Depending on their pigmentation they cause green or red mass blooms during the melt season. This decreases surface albedo and thus further enhances snow and ice melting. Although the phenomenon of snow algal blooms has been known for a long time, large aspects of their physiology and ecology sill remain cryptic. This study provides the first in-depth and multi-omics investigation of two very striking adjacent green and red snow fields on a glacier in Svalbard. We have assessed the algal community composition of green and red snow including their associated microbiota, i.e., bacteria and archaea, their metabolic profiles (targeted and non-targeted metabolites) on the bulk and single-cell level, and assessed the feedbacks between the algae and their physico-chemical environment including liquid water content, pH, albedo, and nutrient availability. We demonstrate that green and red snow clearly vary in their physico-chemical environment, their microbial community composition and their metabolic profiles. For the algae this likely reflects both different stages of their life cycles and their adaptation strategies. Green snow represents a wet, carbon and nutrient rich environment and is dominated by the algae *Microglena* sp. with a metabolic profile that is characterized by key metabolites involved in growth and proliferation. In contrast, the dry and nutrient poor red snow habitat is colonized by various *Chloromonas* species with a high abundance of storage and reserve metabolites likely to face upcoming severe conditions. Combining a multitude of techniques we demonstrate the power of such complementary approaches in elucidating the function and ecology of extremophiles such as green and red snow algal blooms, which play crucial roles in glacial ecosystems.

## Introduction

Snow algae are poly-extremophilic microalgae that thrive on snow fields and glaciers in polar and alpine regions. They are prolific primary colonizers and producers (Lutz et al., 2014) despite being subjected to a multitude of harsh environmental conditions including low temperatures, high irradiation, freeze-thaw cycles, desiccation, low pH, and broad variation in the levels of nutrients. They have evolved specialized cryogenic adaptations including accumulation of secondary carotenoids to shield the photosystem from excessive irradiation and the formation of robust spores with thick cell walls (Remias et al., 2010b). In spring, when the snow starts to melt, extensive snow algal blooms occur. Depending on the nature and composition of the colored pigments, these blooms cause a green or red (all shades from orange to pink) coloration of the snow. As part of the life cycle and as a mechanism of protection from high irradiation, snow algae can adjust their pigmentation from predominantly chlorophylls (“green snow”) to carotenoids (“red snow”) (Remias et al., 2005). However, it is still unknown whether all green snow undergoes a transition to red snow or whether red and green snow represent two independent phenomena. The coloration causes a darkening of snow surfaces, which in turn decreases surface albedo and eventually may speed up melting processes (Thomas and Duval, 1995; Yallop et al., 2012; Benning et al., 2014; Lutz et al., 2014; Lutz et al., unpublished data).

First described by Aristotle (Gentz-Werner, 2007), snow algae have been known for a long time and they have been studied in many cryospheric settings including Svalbard (Mueller et al., 2001; Leya et al., 2004; Stibal et al., 2007; Lutz et al., unpublished data), Iceland (Lutz et al., 2015), Alaska (Takeuchi, 2002), Greenland (Lutz et al., 2014), the Himalayans (Yoshimura et al., 2006), the Rocky Mountains (Thomas and Duval, 1995), Antarctica (Fujii et al., 2010; Remias et al., 2013), and the European Alps (Remias et al., 2005). Most of the ‘true’ snow algae belong to the *Chlamydomonadaceae* (*Chlorophyta*). Dramatic morphological changes during their life cycles make an unambiguous species identification by microscopy very challenging and susceptible to misassignments. Therefore, the most described taxa *Chlamydomonas nivalis* and *Chloromonas nivalis* are actually polyphyletic and must be treated as collective taxa (Leya et al., 2004; Matsuzaki et al., 2015).

Many studies have addressed various aspects of snow algal ecology and physiology (Kol, 1968; Hoham and Duval, 2001; Takeuchi, 2002; Leya et al., 2004) and targeted individual metabolic groups including pigments (Remias et al., 2005; Leya et al., 2009) and fatty acids (Spijkerman et al., 2012). However, a detailed snow algal species characterization as well as their functionality remain cryptic. To our knowledge, germination of mature red snow algal spores and the replication of a full snow algal life cycle from trophic stages to spores under controlled laboratory conditions have so far been unsuccessful. Therefore, a better understanding relies heavily on a comprehensive collection and evaluation of field samples, which in turn are not always clearly interpretable.

Nevertheless, combining various ‘omic’ and metabolite analyses on such samples may help further elucidate their life cycle. Metabolites are the end product of cellular biochemical processes and therefore the ultimate response of an organism to their environment (Jamers et al., 2009). As such, the complement of metabolites within an organism – its metabolome – may provide insights into potential stress factors in the environment (Viant, 2007). For instance, secondary metabolites, which are small, polar molecules not directly related to growth, development or reproduction, are key to algal survival and thriving. Secondary metabolites are often multifunctional, commonly acting as antioxidants. Such compounds act to either inhibit the generation of reactive oxygen species or to quench them when they do form. This helps to maintain the cell’s redox homeostasis (Kutchan and Dixon, 2005; Vickers et al., 2009; Agati et al., 2012; Ramel et al., 2012). Individual groups of metabolites have been targeted in snow algae including pigments (Bidigare et al., 1993; Remias et al., 2005; Leya et al., 2009), fatty acids (Řezanka et al., 2008; Spijkerman et al., 2012), and phenolics (Duval et al., 1999). However, the used targeted metabolic analyses were restricted to a few metabolites and as such do not have the high-throughput capacity of the so-called ‘omics’ techniques. Despite the increasing power and resolution of these new techniques in recent years, they have not thus far been applied to cryophilic snow algae. One reason could be the lack of appropriate reference genomes as currently, the closest fully sequenced green algal strain is *Chlamydomonas reinhardtii* (Merchant et al., 2007), a model organism for freshwater *Chlorophyta* adapted to mesophilic temperatures and hence not sharing common cryogenic adaptations.

**FIGURE 1:**
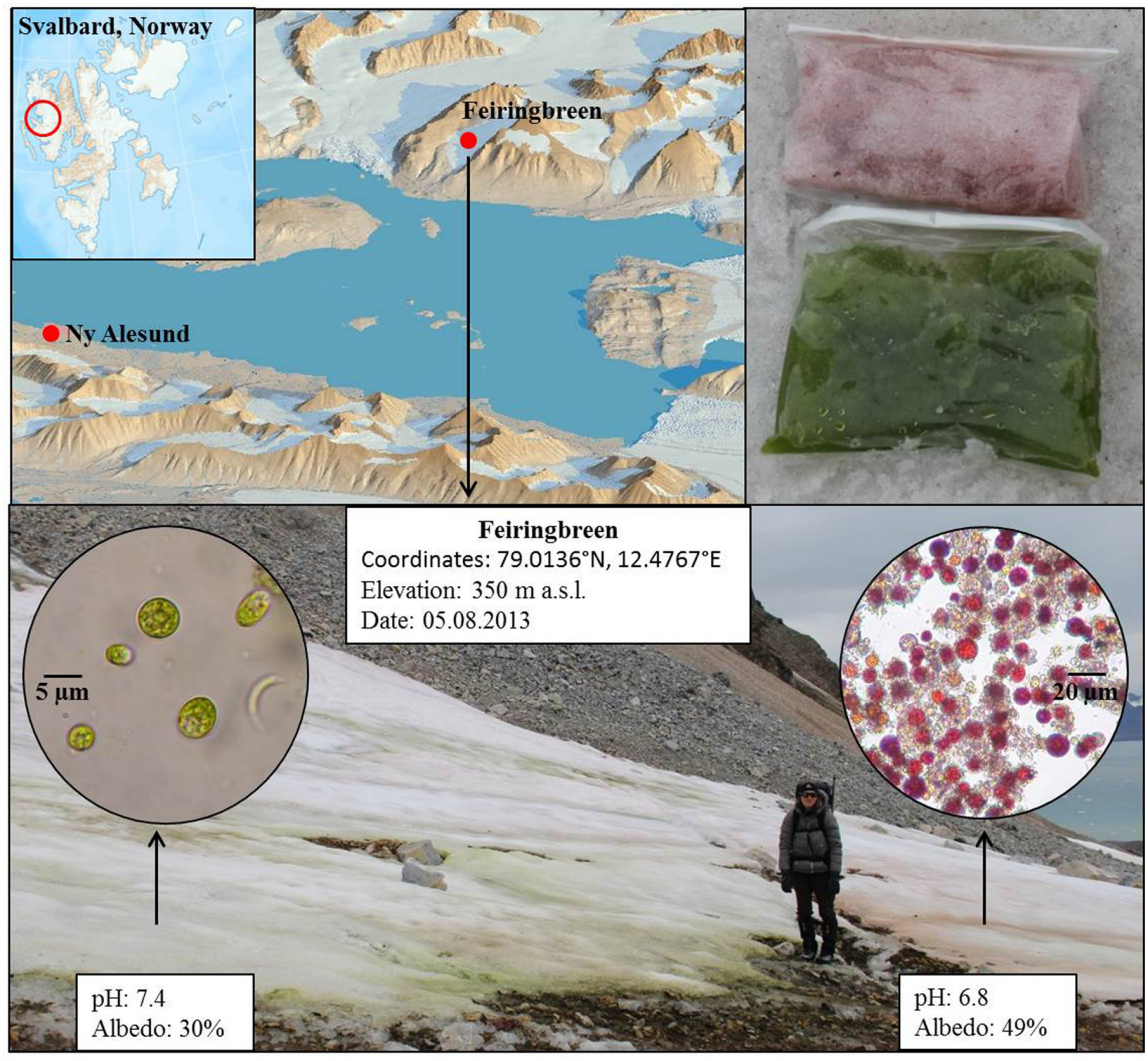
**Map (**top**; left) showing the sample location on Feiringbreen in Svalbard, bags with sampled green and red snow (**top**; right) and image of the site with superimposed microscopic images of the snow algal cells **(bottom)****. Shown are also coordinates and in field measured pH and albedo values for each snow type.

To close this gap we explored in this study the questions whether (a) the formation of green and red snow are linked or independent phenomena, (b) if and how the life stages of red and green snow algae differ, and (c) what the potential feedbacks are between the presence of algae and their physico-chemical environment, which is crucial for understanding the potential importance of glacial biomes for the export of metabolites to downstream ecosystems. We targeted specific metabolic groups including pigments, fatty acids and carbohydrates, and complemented these analyses through detailed metagenomics and metabolomics analyses and quantified the functional gene inventory and respective metabolomes of green and red snow communities. These bulk analyses were also complemented by targeted single cell synchrotron infrared spectroscopic analyses to determine functional groups in individual snow algal cells for the first time. Such targeted or non-targeted techniques inherit their own advantages and challenges. With this contribution we promote the power of a combined application of a multitude of different techniques to help elucidate the snow algal phenomenon in two adjacent very striking green and red snow algae fields on a glacier in Svalbard (**Figure 1**). Understanding snow algal diversity and functioning will not only help us obtain a better understanding of the communities themselves but also help us assess the environment they live in and the potential changes this environment may undergo, particularly in light of the increased melting due to the fast changing Arctic climate.

## Material And Methods

### Field Site, Sampling, and Measurements

Two adjacent, very striking green and red snow algae fields were sampled on Feiringbreen in Svalbard on the 8th of August in 2013 (**Figure 1**). The snow fields were found next to a cliff in the close vicinity of bird rookeries. The intensely green colored snow was very wet and formed a layer of about 10 cm in thickness, whereas the red (nearly pink) coloration was equally striking, but could only be found in the top centimeter of much drier snow. Often the red snow was separated from the green snow by between 1 and up to 30 cm of white, clean snow (without macroscopically visible colored cells or debris), yet some sections of the red snow were immediately adjacent to the green snow (**Figure 1**).

All sampling and field measurements as well as several of the analytical approaches have previously been described in full (Lutz et al., 2014, 2015). Here we only summarize these methods and give full details of newly used methods. In brief, at each sampling site we measured pH, conductivity and temperature with a daily calibrated meter (Hanna instruments, HI 98129) prior to sampling. Photosynthetic active radiation (PAR) and UV irradiation, as well as surface albedo (400–700 nm range), were measured using a radiometer (SolarLight, PMA2100). Samples were collected in sterile centrifuge tubes or sterile *Whirl-Pak*^®^ bags (**Figure 1**) and in pre-ashed glass jars (450°C > 4 h) for organic analyses. All samples were slowly melted at room temperature within ∼6 h of collection and processed and preserved (e.g., filtered, acidified) for further analyses within ∼8 h following collection. Samples for DNA and all metabolite analyses were flash-frozen in liquid nitrogen and stored at –80°C until analyzed. All inorganic samples (both filtered aqueous fractions and particulates) were stored cold (4°C) and in the dark.

### Aqueous and Particulate Geochemical Characterizations

Aqueous analyses were carried out by Ion Chromatography (IC, Dionex, anions) and by inductively coupled plasma mass spectrometry (ICP-MS, Agilent, cations). We used a total organic carbon analyser (Shimadzu, TOC 5000A) for dissolved organic carbon (DOC) measurements, and a segmented flow-injection analyses (AutoAnalyser3, Seal Analytical) for analyses of dissolved phosphate.

Particulates, separated from solutions through filtration were analyzed for total carbon (TC), total nitrogen (TN), total sulfur (TS), as well as δ^15^N and δ^13^C, by pyrolysis at 1500°C (Vario Pyro Cube, Elementar Inc.) followed by mass spectrometry (Isoprime Mass Spectrometer). Particulate phosphorus was extracted by ashing of the samples at 550°C for 2 h and incubating in 1 M HCl for 16 h according to extraction step V in Ruttenberg et al. (2009). The mineralogy of the particulates was determined by X-ray diffraction (XRD, Bruker D8).

### Algal Abundance and Biomass Evaluations

Algal cells were imaged on a Leica DM750 microscope equipped with a 63× objective and counted with a hemocytometer in triplicates. For cell size analyses, 100 cell diameters per sample were measured in ImageJ. Cell volumes were calculated assuming a perfect spherical shape (*V* = 4/3^∗^π^∗^r^3^) and total algal biomass was calculated using the average cell volume and cell abundance.

### Amplicon and Metagenome Sequencing

Total DNA was extracted using the PowerSoil^®^ DNA Isolation kit (MoBio Laboratories). 16S rRNA genes were amplified using bacterial primers 27F (5′-AGAGTTTGATCMTGGCTCAG) and 357R (5′-CTGCTGCCTYCCGTA) (tagged with the Ion Torrent adapter sequences and MID barcode) spanning the V1–V2 hypervariable regions. 18S rRNA genes were amplified using the eukaryotic primers 528F (5′-GCGGTAATTCCAGCTCCAA) and 706R (5′-AATCCRAGAATTTCACCTCT) (Cheung et al., 2010) (tagged with the Ion Torrent adapter sequences and MID barcode) spanning the V4-V5 hypervariable region. Polymerase chain reactions (PCR) were performed using Platinum^®^ PCR SuperMix High Fidelity according to manufacturer’s protocols. Initial denaturation at 95°C for 5 min was followed by 30 cycles of denaturation at 95°C for 30 s, annealing at 60°C for 30 s and elongation at 72°C for 30 s. Final elongation was at 72°C for 7 min. Archaeal 16S rRNA genes were amplified following a nested PCR approach. The first PCR reaction was carried out using primers 20F and 915R. Initial denaturation at 95°C for 5 min was followed by 35 cycles of denaturation at 95°C for 30 s, annealing at 62°C for 30 s and elongation at 72°C for 180 s. Final elongation was at 72°C for 10 min. The PCR product was used as template for the second PCR reaction with primers 21F (5′-TCCGGTTGATCCYGCCGG) and 519R (5′- GWATTACCGCGGCKGCTG) (tagged with the Ion Torrent adapter sequences and MID barcode) spanning the V1–V2 hypervariable region. Initial denaturation at 95°C for 5 min was followed by 30 cycles of denaturation at 95°C for 30 s, annealing at 60°C for 30 s and elongation at 72°C for 30 s. Final elongation was at 72°C for 7 min. All PCRs were carried out in triplicates to reduce amplification bias and in reaction volumes of 1 × 25 μl and 2 × 12.5 μl. All pre-amplification steps were carried out in a laminar flow hood with DNA-free certified plastic ware and filter tips. The pooled amplicons were purified with AMPure XP beads (Agencourt^©^) with a bead to DNA ratio of 0.6 to remove nucleotides, salts and primers and analyzed on the Agilent 2100 Bioanalyser (Agilent Technologies) with the High Sensitivity DNA kit (Agilent Technologies) and quality, size and concentration were determined. Sequencing was performed on an Ion Torrent Personal Genome Machine using the Ion Xpress^TM^ Template Kit and the Ion 314^TM^ or Ion 316^TM^ chips following manufacturer’s protocols. The raw sequence data was processed in QIIME (Caporaso et al., 2010). Barcodes and adapter sequences were removed from each sequence. Filtering of sequences was performed using an average cutoff of Q20 over a 350 bp range. Reads shorter than 200 bp were removed. OTUs were picked *de novo* using a threshold of 97% identity. Taxonomic identities were assigned for representative sequences of each OTU using the reference databases Greengenes for bacteria and archaea. The Silva database (DeSantis et al., 2006; extended with additional 223 sequences of cryophilic algae kindly provided by Dr Thomas Leya from the CCCryo – Culture Collection of Cryophilic Algae, Fraunhofer IZI-BB) was used for eukaryotes. Data were aligned using PyNAST and a 0.80 confidence threshold. Singletons were excluded from the analysis. Bacterial sequences matching plant plastids were removed from the data set prior to further analysis. Eukaryotic sequences matching *Chloroplastida* were pulled out of the data set and stored in a separate OTU table. In order to focus upon algal diversity, sequences matching *Embryophyta* (e.g., moss, fern) were removed from the data set. For archaea, sequences matching bacteria were removed.

Metagenome libraries were constructed using the Ion Plus Fragment Library^TM^ and the Ion Xpress Barcode Adapter^TM^ kits according to manufacturer’s instructions. The libraries were analyzed on an Agilent 2100 Bioanalyser (Agilent Technologies) with a High Sensitivity DNA kit (Agilent Technologies) to determine DNA quality, size, and concentration. Sequencing was performed on an Ion Torrent Personal Genome Machine using the Ion Xpress^TM^ Template Kit and the Ion 314^TM^ chip following manufacturer’s protocols. The raw sequence data was processed in QIIME (Caporaso et al., 2010). Barcodes and adapter sequences were removed from each sequence. Filtering of sequences was performed using an average cut-off of Q20 over a 350 bp range and a minimum read length of 200 bp. Sequence reads were assigned to protein references sequences in the IMG database using *blat* (Kent, 2002). The KEGG Orthology (KO) system was used to derive major functional categories for the annotated genes. Sequences have been deposited to the European Nucleotide Archive (ENA) under accession number PRJEB11474.

### Non-targeted Metabolome Analyses

Samples were centrifuged at 20,000 rpm to pellet algal cells. Pellets were ground to a powder in liquid nitrogen. Biphasic extractions of algal metabolites were prepared as per the methods of Overy et al. (2005) in 750 μl of a sterile methanol:chloroform:water/6:2.5:1 mix for 60 min on ice. 400 μl of sterile water were added and extracts were vortexed and centrifuged at 12,000 rpm for 2 min to produce biphasic extractions. 200 μl of the aqueous/polar phase of each metabolite extract was diluted with 200 μl of methanol and 400 μl 0.1% formic acid and analyzed by direct injection ToF-LC-MS (QStar Elite System, Applied Biosystems, Waltham, MA, USA). Broad range spectra for mass numbers 50–1000 Da were collected over 240 cycles (each cycle lasting 0.9998 s) in positive ion mode using the following instrument settings: resolution of 4000, GS1 at 27.0, CUR at 20.0, and IS of 3500.0. Source temperature was 100°C and cone gas flow rate was maintained at 10 μl min^-1^. Samples were run in triplicate and the resulting spectra combined using the Analyst QS 2.0 (Applied Biosystems, Waltham, MA, USA) software. Mass numbers (or mass to charge ratios, *m*/z) were rounded into 0.01 Da bins and the relative abundance (Total Ion Count – %TIC) for each mass number within that bin summed (Overy et al., 2005; Field and Lake, 2011). All data processing was carried out using in-house software (Burrell and Cameron, unpublished) based upon the binning procedures of Overy et al. (2005).

Metabolic profiles were compared using the OPLS-DA method and the Simca-P multivariate data analysis software (Umetrics, Sweden) using binned mass numbers as the primary variable and sample type as the observational variable. Discriminatory *m/z* values with an *R*^2^ close to 1.0 were assigned identities and metabolic pathways using the online database Biocyc^1^ and the Kyoto Encyclopedia of Genes and Genomes (KEGG)^2^, using the *Chlamydomonas reinhardtii* reference library wherever possible.

### Targeted Bulk Metabolite Analyses

To determine the bulk carotenoid and chlorophyll contents in the samples, high pressure liquid chromatography (HPLC) and a modified carotenoid/chlorophyll specific extraction protocol (Remias and Lutz, 2007) were used. Cells were disrupted by shock freezing in liquid nitrogen for 10 min followed by grinding using a Teflon^®^ mortar and pestle. The resulting powder was re-suspended in 1 mL of dimethylformamide (DMF) and 1.0 mm glass beads and horizontally shaken on a laboratory shaker (MoBio Vortex Genie 2) at maximum speed (3000 rpm) for 10 min followed by centrifugation for 5 min at 10 000 rpm. The supernatant was separated from the debris by filtering through a 0.45 μm Teflon^®^ filter and the filtrate was mixed with methanol (25 vol %). Extracted samples were analyzed immediately on an Agilent Technologies 1200 Infinity HPLC instrument with a gradient pump, an autosampler, a variable wavelength detector and ODS Hypersil column (250 × 4.6 mm; 5 μm particle size). Two solvents were used: solvent A consisted of a mixture of acetonitrile/water/methanol/hexane/tris buffer at ratios of 80:7:3:1:1, while solvent B was a mix of methanol and hexane at a ratio of 5:1. The HPLC was run at a flow rate of 1 mL min^-1^ and with an injection volume of 25 μL. Spectra were recorded from 200 to 800 nm and chromatograms were quantified at 450 nm for carotenoids and 660 nm for chlorophyll a and b. Run time was 60 min and the protocol required a 15 min run with 100% of solvent A followed by a linear gradient from 100% solvent A to 100% solvent B between 32 and 45 min and finally with 15 min of column re-equilibration through a 5 min linear gradient from solvent B back to 100% solvent to A, followed by a further column conditioning with 100% solvent A for 10 min. The following commercially available standards were used for peak identification and pigment quantifications: chlorophyll a, chlorophyll b (Sigma), violaxanthin, neoxanthin, antheraxanthin, lutein, β-carotene, *trans*-astaxanthin, and *cis*-astaxanthin (Carotenature).

Fatty acids were extracted from the particulates according to the method described by Wacker and Martin-Creuzburg (2007). Briefly, 20 ng of internal standard (tricosanoic acid methyl ester) were added to each sample, before ultrasonic extraction using dichloromethane:methanol (2:1 v:v), followed by centrifugation to remove particulates and evaporation of solvent from the supernatant. Fatty acids were transesterified by adding methanolic HCl to the dried extract and heating at 60°C for 20 min. After cooling, fatty acid methyl esters were extracted in isohexane, the solvent was removed under nitrogen and the sample resuspended in isohexane for analysis. Analysis of fatty acid methyl esters was carried out using a Trace 1300 gas chromatograph with flame ionization detector (Thermo Scientific, Hemel Hempstead, UK), equipped with a non-polar-fused silica capillary column (CPSil-5CB, 50 m × 0.32 mm × 0.12 mm, Agilent Technologies, USA). Samples (1 μl) were injected in splitless mode, with the injector maintained at 200°C. Carrier gas was helium, and a constant flow rate of 1.5 ml/min was used. The following temperature program was used: initial temperature 40°C, rising to 140°C at 20°C min^-1^, then rising to 240°C at 4 min^-1^, holding at 240°C for 5 min. Fatty acid methyl esters were identified by comparison of retention times with those of reference compounds (37 Component FAME Mix, Supelco, PA, USA) and by gas chromatography/mass spectrometry. Gas chromatography/mass spectrometry analyses was carried out using the gas chromatograph and column previously described, with identical operating conditions, but coupled to an ISQ mass spectrometer (Thermo Scientific, Hemel Hempstead, UK). The transfer line and the ion source were maintained at 300°C. The emission current was set to 50 mA and the electron energy to 70 eV. The analyzer was set to scan the mass to charge ratios between 50 and 650 with a scan cycle time of 0.6 s.

Carbohydrate contents and concentrations were determined on a Dionex ICS-3000 Ion Chromatography system (Sunnyvale, CA, USA). The carbohydrates fucose, rhamnose, arabinose, galactose, glucose, xylose/mannose, fructose/sucrose, ribose, and lactose were separated isocratically on a CarboPac PA20 column (3 mm × 150 mm), after passing through a CarboPac PA20 guard column (3 mm × 30 mm). Fructose/sucrose and xylose/mannose are co-eluting and hence are reported together.

The relative abundance of functional groups corresponding to proteins, lipids, and carbohydrates was evaluated on the bulk particulate samples after deposition of a dried sample aliquot on a single pass diamond window of an Attenuated Total Reflection cell of a Fourier transform infrared spectroscope (FTIR, A2 Technology Microlab). For each bulk particulate sample 1064 spectra collected at a resolution of 4 cm^-1^ over the mid infrared region between 650 and 4000 cm^-1^ were co-added.

### Single-cell Micro-spectroscopy

Micro-analyses were carried out at the Multimode infrared imaging and micro-spectroscopy (MIRIAM) beamline, B22 at the Diamond Light Source (UK). Individual snow algal cells that were thawed and deposited on ZnSe windows were imaged and analyzed in transmission mode by FTIR spectroscopy. A Bruker FTIR spectrometer interlinked with the synchrotron-light (Benning et al., 2004) and a microscope was used to collect images and spectra via a broadband MCT detector, a ×36 objective and a ×36 condenser. We collected data either using a 20 μm × 20 μm aperture or a 6 μm × 6 μm aperture. Spectra were acquired over the mid-infrared range between 4000 and 650 cm^-1^ and at each point/pixel 512 spectra were co-added. All data were processed in Opus (V7.2). Functional group values for individual cells were derived from peak areas under the CH_2_–CH_3_–CH lipid/protein bands (∼3100–2800 cm^-1^), the C–O of the ester lipid band (∼1720 cm^-1^), the main protein bands (1700–1600 cm^-1^ for amide 1) and 1600–1500 cm^-1^ for amide 2) and the carbohydrate bands (between 1200 and 930 cm^-1^). Area ratios for total proteins (1700–1500 cm^-1^) over C–H lipids (3050–2800 cm^-1^) and proteins (1700–1500 cm^-1^) over C–O ester lipids (1850–1700 cm^-1^) were calculated. We quantified the functional groups in 4 green and 12 red single cells and collected on each cell between 12 and 64 single spectra. It is worth noting that we analyzed single spherical cells in transmission mode and thus all spectra represent average intensities through the spheres.

## Results

### Algal Biomass

The intense green snow reached a layer thickness of about 10 cm and the snow was very wet, whereas the red snow formed only a thin (∼1 cm) layer on top of much drier snow. In terms of algae the green snow consisted primarily of small, mostly flagellated cells (**Figure 1**) with an average diameter of 11 μm and cell volume of ∼700 μm^3^ (**Table 1**). The variably reddish colored algal cells in the red snow consisted mainly of spores with an average diameter of 17 μm and cell volume of ∼2600 μm^3^. Algal cell numbers were an order of magnitude higher in green snow (6 × 10^6^ mL^-1^) compared to red snow (2 × 10^5^ mL^-1^) and overall biomass was an order of magnitude higher in green snow (∼450 mm^3^ L^-1^, as opposed to ∼50 mm^3^ L^-1^) (**Table 1**).

**Table 1:** Cell counts, average cell sizes, and overall biomass for green and red snow.

	Green snow	Red snow
Cell counts [mL^-1^]	640,625	20,313
Average cell diameter [μm]	11.2 ± 2.5	17.1 ± 2.6
Average cell volume [μm^3^]	697	2571
Biomass [mm^3^ L^-1^]	446	52

### Physico-geochemical Characteristics of Solutions and Particulates

In both red and greed snow patches the temperature (0°C) and conductivity (<2 μS/cm^2^) were similar but the pH differed by ∼0.6 pH units with a higher value in the green snow (7.4) compared to red snow (6.8).

In terms of aqueous compositions, most major (e.g., Na, K, Ca, Mn), minor (e.g., Fe, Al) and trace elements (e.g., Co, Cr, Cu, Ni) were between 1 and 3 orders of magnitude higher in concentration in green snow compared to red snow (**Table 2**). DOC and PO_4_ were 10 times higher in green snow, whereas NO_3_ and SO_4_ were high in the green snow but below the limit of detection in red snow. Cl was the only element that was present in red but not in green snow. The concentrations of S analyzed by ICP-MS match those of S–SO_4_, whereas the concentrations of P (also ICP-MS) were much higher than those analyzed by IC as P–PO_4_ (**Table 2**).

**Table 2:** Organic (DOC) and inorganic aqueous chemical data for green and red snow.

	Green now	Red snow
DOC [μM]	1192	67
PO_4_^3-^ [μM]	8.63	0.53
NO_3_^-^	296	<
SO_4_^2-^	31421	<
Cl^-^	<	277
Al	6.2	5.8
Ba	7.2	7.2
Ca	22900	2660
Cd	0.1	0.2
Co	0.8	<
Cr	0.1	<
Cu	1.1	0.2
Fe	10.6	6.3
K	5620	55
Mg	3880	390
Mn	41.0	2.6
Na	720	185
Ni	1.7	0.2
P	1050.0	<
Pb	0.2	0.1
S	11400	49
Si	160	40
Sr	26.0	2.6
Zn	1.5	2.1

*DOC and PO4 are in μM, whereas all other compounds are in ppb. NO^3-^, SO_4_^2-^, and Cl^-^ all determined by IC, all others analyzed by ICP-MS; limit of detection (LOC, <) for IC: NO_3_^-^= 96 ppb, Cl^-^= 72 ppb, SO_4_^2-^ = 121 ppb, LOD’s for ICP-MS: Al, Ba, Co, Cr, Cu, Fe, Mg, Ni, Si, Sr, Zn = 0.1 ppb; Cd, Mn, Pb = 0.01 ppb; Ca, Na = 1 ppb; K,P,S = 10 ppb*.

In the particulates, the TC content per dry weight was similarly high in both samples (green: 35.4%, red: 33.4%) while TN was higher in green snow (6.7%, as opposed to 1.9% in red snow). This led to a C/N ratio (**Table 3**) for green snow (5.3) near the optimal Redfield ratio (6.6), whereas the ratio for red snow was much higher (17.7). The C/P ratio for red snow was also seven times higher (754) than optimal Redfield conditions (106), while the δ^13^C value was more negative in red snow (–29.73‰) compared to green snow (–27.67‰).

**Table 3:** Total carbon (TC), total nitrogen (TN), total phosphorus (TP), and total sulfur (TS) (all based on % of dry weight of sample), nitrogen and carbon isotope values, and particulate C/N, C/P, and N/P ratios calculated from TC, TN, and TP values.

	Green snow	Red snow
Total C [%]	35.39	33.44
Total N [%]	6.73	1.89
Total P [%]	n.s.	0.04
Total S [%]	0.58	0.19
C/N	5.26	17.69
C/P	–	743.82
N/P	–	42.05
δ^15^N [‰]	n.s.	0.73
δ^13^C [‰]	-27.67	-29.73

*n.s. = not enough sample for analyses*.

The prevailing mineralogy in both particulate samples were quartz, calcite, dolomite, chlorite, and muscovite corresponding with the prevailing geological units that are surrounding Feiringbreen and that consist primarily of late Palaeozoic (mostly Carboniferous) marbles and mica shists of the Kongsfjorden group (Harland, 1997).

### Amplicon and Metagenome Sequencing

**FIGURE 2:**
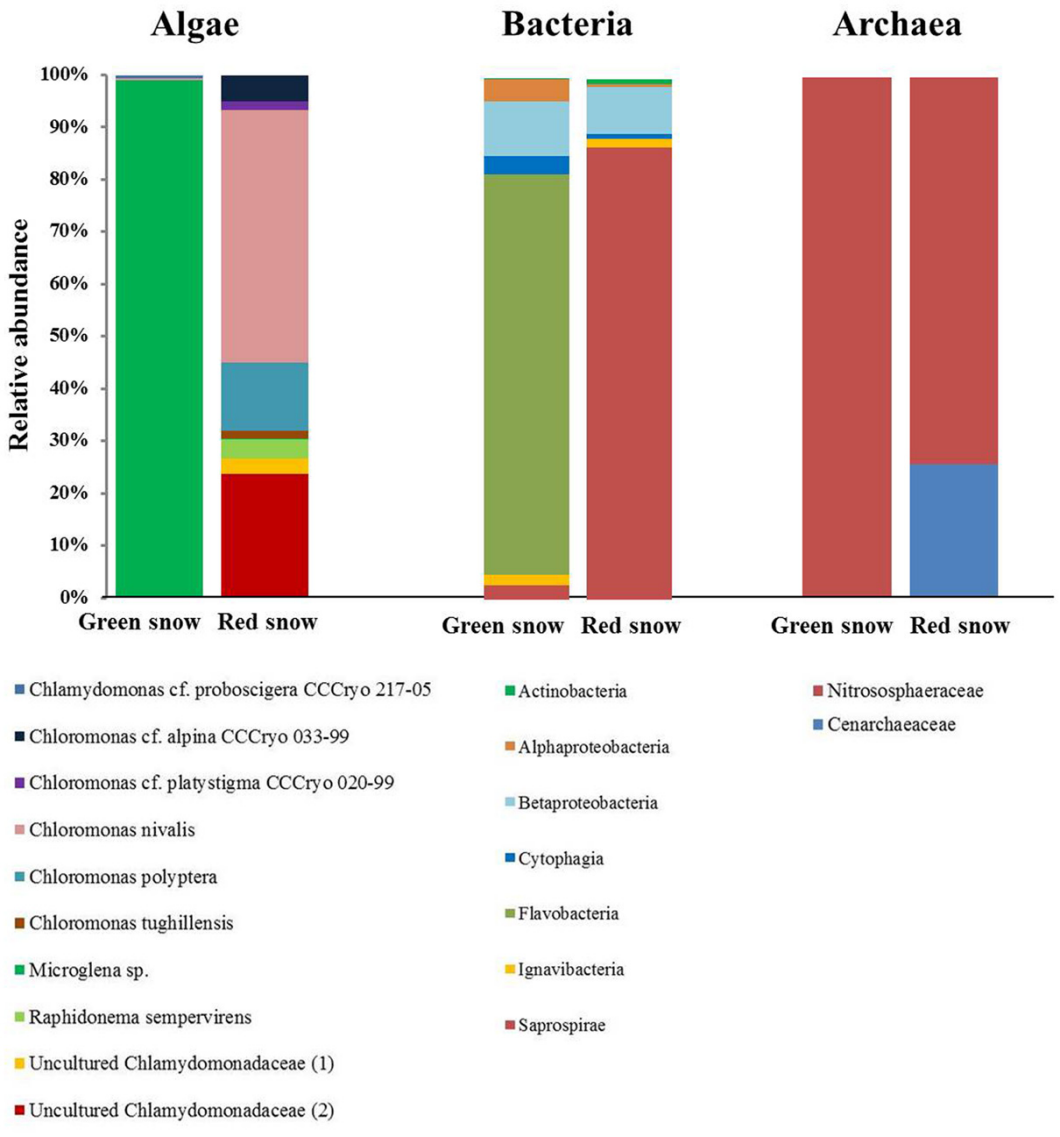
**Algal (18S rRNA), bacterial (16S rRNA), and archaeal (16S rRNA) community composition of green and red snow, derived from OTU clustering at 97% similarity**.

The two samples showed different algal species compositions (**Figure 2**, Supplementary Table S1). In the green snow *Microglena* sp. made up 99% of the species composition, whereas in red snow several *Chloromonas* and uncultured *Chlamydomonadaceae* species contributed to a relatively higher species diversity. The most relative abundant species were *Chloromonas nivalis* (48%), an uncultured *Chlamydomonadaceae* (labeled “2”, 24%), *Chloromonas polyptera* (13%), *Chloromonas cf. alpina* (5%), and *Raphidonema sempervirens* (4%).

For bacteria, *Bacteriodetes* followed by *Proteobacteria* were the most relative abundant phyla in both samples, yet at the class level the bacterial communities were more markedly different (**Figure 2**, Supplementary Table S2) with green snow being dominated by *Flavobacteria* (77%), followed by *Betaproteobacteria* (10%), whereas red snow was dominated by *Saprospirae* (87%), followed by *Betaproteobacteria* (9%). It is important to note that *Saprospirae* is an obsolete class and on the family level all sequences were assigned to the *Chitinophagaceae* (Supplementary Table S2).

The archaeal species composition was made up by only one taxon in green snow, the *Nitrosophaeraceae* (100%), whereas in red snow *Nitrosophaeraceae* were most abundant (74%), followed by *Cenarchaeaeceae* (26%) (**Figure 2**, Supplementary Table S3).

There were no major differences between the two metagenomes in terms of main functional categories for the annotated genes based on KO (Supplementary Figure S1; Supplementary Table S4). Genes thought to be involved in carbohydrate metabolism were most abundant (green snow: 12.7%, red snow: 15.2%), followed by amino acid metabolism (green snow: 11.2%, red snow: 9.6%), and energy metabolism (green snow: 8.9%, red snow: 9.2%). Genes involved in carotenoid and fatty acid biosynthesis were below 1% in both samples.

### Non-targeted Metabolome Data

**FIGURE 3:**
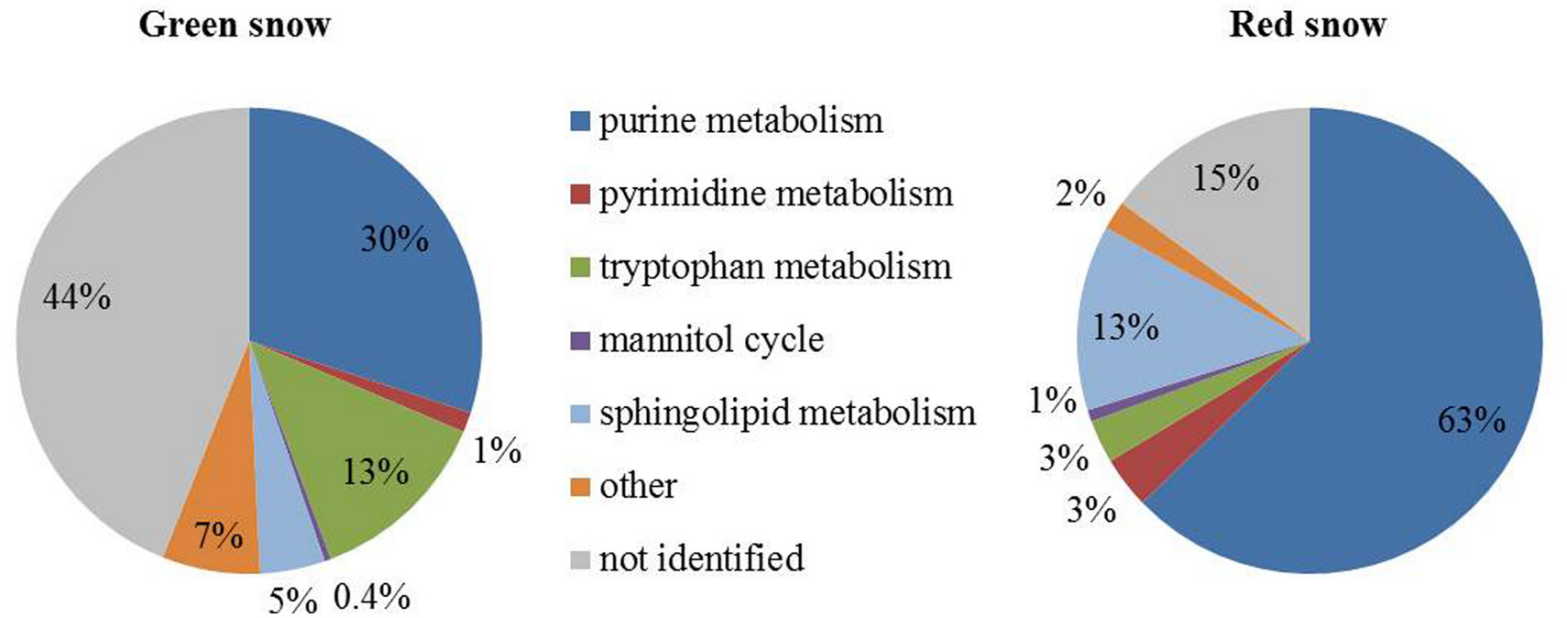
**Pie charts showing metabolomics data grouped into main metabolic pathways in green and red snow**.

Twenty-nine mass numbers (*m/*z values) were assigned putative identities and pathways, leading to the identification of 85% of all metabolites in red snow and 56% in green snow (**Table 4**; **Figure 3**). Among these *m/z* 364, corresponds to 5-formamido-1-(5-phospho-D-ribosyl)-imidazole-4-carboxamide, which is a key intermediate compound in several plant secondary metabolite biosynthesis pathways. This compound made up 23.6% of the metabolites in the green snow and was the dominant compound in the red snow (61.2%). Sphinganine 1-phosphate (*m/z* 380), which is part of the sphingolipid metabolic pathway, was the second most relative abundant metabolite in red snow (12.9%), but less represented in green snow (4.5%). Orotidylic acid (*m/*z 365), involved in various metabolic pathways including the uridine monophosphate biosynthesis within the pyrimidine metabolism (Supplementary Figure S2), was the third most abundant compound in red snow (3.6%), while in green snow it was just of minor abundance (1.3%) (**Table 4**). In green snow, the mass number *m/z* 276, matching 5-amino-6-D-ribitylaminouracil, a key metabolite involved in several metabolic pathways including riboflavin metabolism (5.7%) was six times more abundant compared to red snow (**Table 4**). Similarly, the mass numbers *m/*z 174 likely to be indole-3-acetate (IAA), also known as auxin and involved in tryptophan metabolism (9.2%), and *m/z* 202 corresponding to indole-3-pyruvate (3.5%), another tryptophan pathway metabolite (Supplementary Figure S3), were also between 2 and 11 times more abundant compared to red snow (**Table 4**).

**Table 4:** Main metabolic compounds with mass numbers, their abundance relative to the total metabolites ingreen and red snow, and candidate metabolites and pathways corresponding to the mass numbers.

Mass number (m/z)	Green snow [%]	Red snow [%]	Candidate metabolite	Pathway
114	0.63	0.60	Fumarate	Purine metabolism
135	1.94	0.09	Adenine	Purine metabolism
139	0.01	0	Carbamyl-phosphate	Pyrimidine metabolism
155	0.06	0.03	Orotate	Pyrimidine metabolism
157	0.07	0.01	Dihydro-l-orotate or (*S*)-dihyrdoorotate	Pyrimidine metabolism
159	0.01	<0.01	Indole-acetaldehyde	Tryptophan metabolism
174	9.18	0.78	Indole-3-acetate (IAA; auxin)	Tryptophan metabolism
175	0.21	0.02	diphosphate	Tryptophan and pyrimidine metabolism
180	0	0.01	Keto-D-fructose or β-D-fructofuranose	Mannitol cycle
182	0.13	0.03	D-sorbitol or D-mannitol	Sorbitol biosynthesis or mannitol cycle
202	3.53	1.81	Indole-3-pyruvate	Tryptophan metabolism
204	0.16	0.46	Tryptophan	Tryptophan metabolism
246	0.67	0.93	(*RS*)-phospho-3-sulfolactate	Coenzyme M biosynthesis I
258	0.10	0.04	β-D-glucose 6-phosphate or β-D-fructofuranose 6-phsosphate	Various
260	0.32	0.78	D-mannitol 1-phosphate	Mannitol cycle
267	3.84	0.32	Adenosine	Purine metabolism
276	5.70	0.93	5-amino-6-(D-ribitylamino)uracil	Various
322	0	<0.01	Uridine-5′-monophosphate	Pyrimidine metabolism
336	0	0.01	Fructose 1,6-bisphosphate	Various
361	0	0.49	Guanosine 5′-phosphate	Purine metabolism
364	23.57	61.25	5-formamido-1-(5-phospho-D-ribosyl)-imidazole-4-carboxamide	Purine metabolism
365	1.34	3.59	Orotidine 5′-phosphate (orotidylic acid)	Pyrimidine metabolism
380	4.52	12.87	Sphinganine 1-phosphate	Sphingolipid metabolism
385	0	<0.01	5-phospho-α-D-ribose 1-diphosphate	Pyrimidine and purine metabolism,
440	0.01	0.02	Guanosine-diphosphate	Purine metabolism
Not identified	43.94	14.94		

*Underlined candidate metabolites have previously been identified in *Chlamydomonas reinhardtii**.

Metabolites potentially involved in purine metabolism were highest in green (30%) and red snow (63%) (**Figure 3**; Supplementary Table S5). Compounds involved in sphingolipid metabolism were the second most abundant in red snow (13%, as opposed to 5% in green snow), whereas tryptophan metabolites were the second most abundant in green snow (13%, as opposed to 3% in red snow; **Figure 3**; Supplementary Table S5).

### Targeted Bulk Metabolites

Chlorophylls were the most abundant pigments in green snow (71%), followed by primary carotenoids (27%), and only traces of secondary carotenoids (5%). In contrast, the pigment composition in red snow was dominated by secondary carotenoids (92%), with only traces of chlorophylls (4%) and primary carotenoids (4%) (**Table 5**).

**Table 5:** Pigment composition of green and red snow.

	Green snow	Red snow
Chlorophyll a	13372	5187
Chlorophyll b	14792	4105
Neoxanthin	1048	309
Violaxanthin	1830	632
Antheraxanthin	166	0
Lutein	6823	2004
Zeaxanthin	110	17
*b*-carotene	0	4768
*Trans*-astaxanthin	0	6433
*Cis*-astaxanthin	0	783
*Trans*-astaxanthin mono esters	1055	148475
*Cis*-astaxanthin mono esters	0	20088
Total astaxanthin diesters	0	37322
**Total chlorophylls**	71%	4%
**Total primary carotenoids**	27%	4%
**Total secondary carotenoids**	3%	92%

*Individual pigments were quantified in μg/L and also reported as total chlorophylls, total primary carotenoids, and total secondary carotenoids in % of total pigments*.

Saturated fatty acids (SFA) identified were mainly C16:0 and traces of C18:0, the monounsaturated fatty acids (MUFA) C16:1 and C18:1, and the polyunsaturated fatty acids (PUFA) C16:4, C18:2, C18:3, and C18:4. In the green snow the SFAs (56%) were most abundant, followed by MUFAs (28%), and PUFAs (4%). In contrast, red snow contained a higher proportion of PUFAs (49%), and only about half as much SFAs (28%) than the green snow while the MUFAs (20%) were similar (**Table 6**).

**Table 6:** Fatty acid composition of green and red snow.

	Green snow (%)	Red snow (%)
C14:0	0	4
C15:0	0	1
C15 branched	4	0
C16:0	53	20
C16:1	13	6
C16:3	0	3
C16:4	0	13
C18:0	3	3
C18:1	15	14
C18:2	0	7
C18:3	0	21
C18:4	4	5
**SFA**	56	28
**MUFA**	28	20
**PUFA**	4	49

*Fatty acid compounds are reported as percentage of total fatty acids. Individual identified fatty acids are reported as well as total saturated (SFA), total monounsaturated (MUFA), and total polyunsaturated fatty acids (PUFA)*.

Free carbohydrates were only abundant in high concentration in the green snow with ribose (246 μg L^-1^), lactose (97 μg L^-1^, fructose–sucrose (14 μg L^-1^) and rhamnose (11 μg L^-1^) being dominant, whereas in red snow most carbohydrate compounds were below 5 μg L^-1^ or below our detection limit (**Table 7**).

**Table 7:** Free carbohydrate analyses, all compounds in μg L^-1^.

	Green snow	Red snow
Fucose	<	<
Rhamnose	10.9	1.2
Arabinose	<	<
Galactose	<	2.3
Glucose	<	2.8
Xylose–Mannose	<	<
Fructose–Sucrose	13.7	2.6
Ribose	245.9	3.0
Lactose	97.0	<

*Detection limits: Fucose < 0.6 μg L^-1^, Rhamnose < 0.5 μg L^-1^, Arabinose < 0.4 μg L^-1^, Glucose < 0.3 μg L^-1^, Xylose–Mannose < 0.4 μg L^-1^, Fructose–Sucrose < 2.4 μg L^-1^, Ribose < 0.9 μg L^-1^, Lactose < 6.8 μg L^-1^*.

The bulk infrared spectroscopy analysis revealed an almost double proportion of proteins in green snow (39%) compared to red snow (20%), yet a slightly increased lipid content in red snow (12%) compared to green snow (7%) (**Table 8**).

**Table 8:** Bulk functional group distribution of green and red snow.

	Green snow (%)	Red snow (%)
Lipids	7.21	11.41
Proteins	38.72	19.64
Carbohydrates	54.08	68.96

*Main functional groups representing the lipids (CH_*2*_ and CH_*3*_ stretching modes between 3050 and 2800 cm^-*1*^), proteins (amide I and II bands at 1700–1500 cm^-*1*^) and carbohydrates (C–O–C, C–O–P, P–O–P ring vibrations between 1204–815 cm^-*1*^) are reported as percentage of total functional groups*.

### Single-cell Functional Groups Data

**FIGURE 4:**
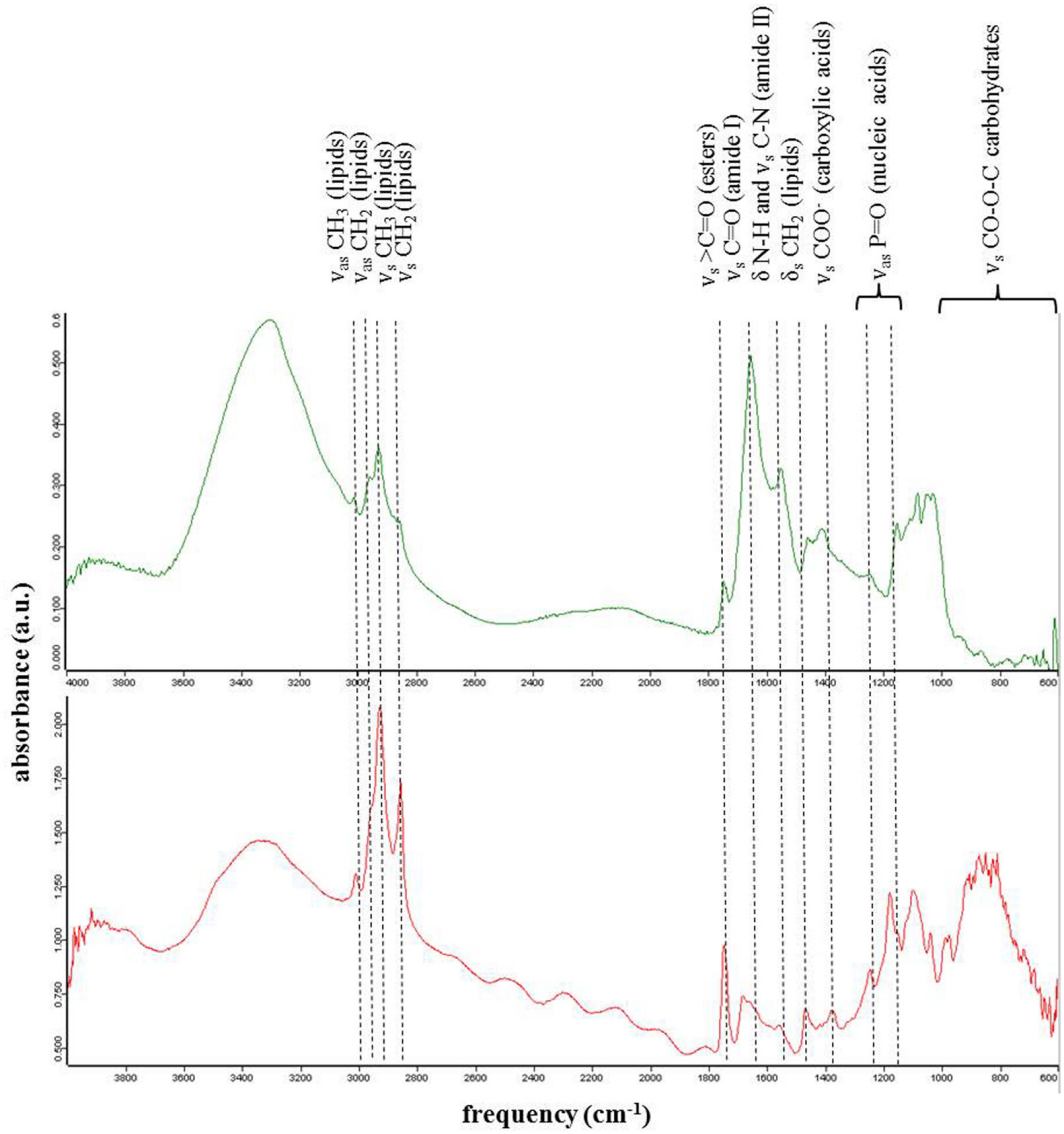
**Representative synchrotron based infrared spectroscopic spectra of single cells in green **(top)** and red **(bottom)** snow**. Green cells were characterized by higher amide peaks compared to red cells that showed higher lipids and ester peaks

From the analyzed single cells using synchrotron infrared micro-spectroscopy we evaluated the protein, lipid, and ester contributions in 124 spectra collected on single green cells and 324 spectra collected on single red cells (**Figure 4**; **Tables 9A,B**). On average the contributions from amides I and II bands corresponding to protein vibrations dominated the spectra in each green algal cell (**Figure 4** top spectrum), whereas ester and lipid functional groups were more abundant in single red algal cells (**Figure 4** bottom spectrum). Ratios of proteins over lipids were on average one order of magnitude higher in green snow algal cells (5.18 ± 0.50) compared to red snow algal cells (0.52 ± 0.42) and proteins over esters were two orders of magnitude higher in green cells (290.56 ± 248.71) compared to red cells (1.76 ± 1.34).

**Table 9:** (A) Ratios of functional group areas corresponding to proteins (amides I and II, 1700–1500 cm^-1^) and lipids (lipids I–IV, 3050–2800 cm^-1^) of single cells in green and red snow derived from synchrotron radiation infrared spectroscopy. **(B)** Ratios of functional groups areas corresponding to proteins (amides I and II, 1700–1500 cm^-1^) and esters (1850–1700 cm^-1^) of single cells in green and red snow derived from synchrotron radiation infrared spectroscopy.

	Green snow			Red snow		
Protein/lipids	Average ±*SD*	Range	*n*	Average ±*SD*	Range	*n*
**(A)**						
Single cell 1	1.43 ± 0.30	0.83–3.05	64	1.07 ± 0.40	0.58–2.46	30
Single cell 2	6.42 ± 1.07	5.20–9.20	36	0.08 ± 0.05	0.02–0.18	25
Single cell 3	8.74 ± 1.40	6.73–11.27	12	0.06 ± 0.02	0.01–0.09	16
Single cell 4	4.12 ± 0.56	3.40–5.19	12	0.28 ± 0.12	0.09–0.48	16
Single cell 5				1.18 ± 0.69	0.43–2.58	25
Single cell 6				0.25 ± 0.09	0.11–0.44	25
Single cell 7				0.35 ± 0.16	0.00–0.74	30
Single cell 8				1.07 ± 0.70	0.06–2.20	16
Single cell 9				0.24 ± 0.09	0.12–0.43	16
Single cell 10				0.40 ± 0.15	0.23–0.85	25
Single cell 11				0.94 ± 1.03	0.00–0.56	64
Single cell 12				0.33 ± 0.36	0.00–1.72	36
Average	5.18 ± 0.50			0.52 ± 0.42		
**(B)**						
Single cell 1	93.73 ± 49.86	52.24–374.18	64	3.29 ± 0.90	2.01–6.57	30
Single cell 2	202.36 ± 205.97	85.36–1097.82	36	0.39 ± 0.18	0.09–0.81	25
Single cell 3	211.23 ± 124.00	89.74–499.58	12	0.36 ± 0.10	0.08–0.46	16
Single cell 4	654.92 ± 733.23	162.37–2193.09	12	0.28 ± 0.12	0.61–3.13	16
Single cell 5				1.69 ± 0.91	0.09–0.48	25
Single cell 6				1.40 ± 0.61	0.49–2.59	25
Single cell 7				4.63 ± 3.94	0.04–13.36	30
Single cell 8				1.07 ± 0.70	0.06–2.20	16
Single cell 9				1.24 ± 0.57	0.61–2.53	16
Single cell 10				2.21 ± 0.47	1.31–3.07	25
Single cell 11				3.15 ± 3.14	0.00–14.41	64
Single cell 12				1.44 ± 1.27	0.00–4.60	36
Average	290.56 ± 248.71			1.76 ± 1.34		

*Reported are single point measurements per cell (*n*), average ratios per cell and standard deviation (SD) and ranges of ratios*.

## Discussion

Despite being localized in close proximity to each other, the two adjacent green and red snow fields described above showed large differences in their physico-chemical environment, their community composition and their metabolic profiles.

### Physico-chemical Environment

Green snow communities are less abundant than red snow communities and have mainly been reported as calcitroph and eurytroph (Kol, 1968) and in favor of relatively high liquid water contents (Fogg, 1967), conditions that are less common for typical snow fields. Such circumstances are usually only found at snow-rock margins (e.g., cliffs) that are often colonized by bird populations, which was also the case in our sampling site on Feiringbreen. The bird rookeries were also evidenced by the much higher nutrient contents (**Table 2**) in the green snow, which was overlaying slightly alkaline carboniferous bedrocks (**Figure 1**).

Previous studies have found a link between pH and snow coloration and this was inferred to be a consequence of the fact that chlorophylls are preferentially synthesized at a higher pH, whereas astaxanthin, the main secondary carotenoid in red snow algae, is favored at lower pH (5.5–6.0) (Czygan, 1970). Similarly, Remias et al. (2013) found green snow in Antarctica with a pH of ∼7.5 and asserted that this was heavily influenced by guano and that the coloration of the snow reached deeper. The green snow in the current study had a higher pH (7.4) than the red snow (6.8) (**Figure 1**), which could be a consequence of carbonate rock buffering through the surrounding bedrocks on which the green snow was found. However, the differing pH could also be the consequence of snow algal activity rather than the cause for their abundance. Such variations in pH could derive from different stages in the snow algal life cycles and their metabolic activities leading to a change in their surrounding environment because the chlorophyll-rich green snow would lead to a net removal of CO_2_ through its photosynthetic activity, and thus an increase in pH. This would also match the assertion of Hoham and Duval (2001) who inferred a relationship between pH and the metabolic state of the snow algal life cycle.

The proximity of the sampled site to a cliff with bird colonies and the high water content likely explains the higher DOC and nutrient concentrations in the green snow (**Table 2**). However, the high Ca, K, and Mg concentrations in green snow (**Table 2**) suggest that not all nutrients are derived from bird droppings but that a large proportion likely stem from the dissolution of carbonate or sulfate minerals in the underlying bedrock. This also explains the high SO_4_^2-^ concentration in green snow derived most likely from dissolution of gypsum, which is a common mineral or cement in Kongsfjorden rocks (Dineley, 1958) although a contribution from bird droppings reflecting a marine food source could also be the cause. The high nutrient concentrations likely induced competition among species leading to a dominance of the green snow community. The mass bloom of green snow algal cells in a high nutrient environment caused a dramatic decrease in surface albedo (30%, **Figure 1**) and therefore increased the heat retention on the snow surface leading to enhanced melting which further increases the liquid water content. In turn, this may have contributed to higher nutrient concentrations as this may aid mineral or bird dropping dissolution.

The discrepancy between the high concentration of total P and P–PO_4_ in green snow suggests a high content of another P species, which is likely to be dissolved organic phosphorus. The high Cl content in red snow but its absence in green snow indicates that the red snow surface layer was affected more or for a longer time by atmospheric inputs (i.e., sea spray) and less so by water–rock interactions with the bedrocks. This matches our observations that the thin layers with red snow algae were not found in contact with the bedrock and the underlying snow was ‘fresher’ i.e., had a shorter deposition residence and metamorphosis time compared to the much wetter green snow.

The high liquid water content in green snow is likely to also act as a protective film against high irradiation. The green flagellates have no thick protective cell walls and are thus much more fragile and sensitive to excessive irradiation. Biflagellates of *Chlamydomonas nivalis* in lab studies have shown to be dramatically affected by UV-B irradiation and showed a high impairment of mobility (Häder and Häder, 1989). UV radiation also inhibits photosynthesis of green snow (*Chloromonas* sp.) by about 85%, compared to an only 25% reduction in red snow (*Chlamydomonas nivalis*) (Thomas and Duval, 1995).

All these factors discussed above suggest that the green and red snow are two independent and not successive phenomena differing in their community composition and life stages. This is also underpinned by the fact that the red snow was much drier and the wet green snow is unlikely to become drier.

The physico-chemical factors including liquid water content, pH and nutrient availability have feedbacks with the snow algae, they likely determine the distribution of the snow algae, yet in turn the snow algae themselves also alter these physical and chemical characteristics after the successful colonization of the snow. The question remains, however, if snow algae at a mature red spore stage have a higher selection advantage than for example the green algal cells lacking this stage. This may be the case according to an investigation by Remias et al. (2010a), who reported that flagellated *Chloromonas nivalis* cells can be found only for a short period of time (and thus most likely are often missed in snow algal studies) because these type of cells have only a very short reproductive phase before they enter the process of spore formation. It is thus not surprising that green snow caused by reproductive cells is often overlooked, because optimal conditions with excess nutrients in snow are not easily maintained, making green snow a rarer and very short-lived phenomenon in nature. This is likely because green flagellated cells are more sensitive to fast changing physico-chemical factors (e.g., water content, pH, nutrients, irradiation) during a melt season. In contrast, the red snow algal spores can cope better with excessive irradiation, desiccation, freeze-thaw cycles and low nutrient concentrations. This may imply that red snow algae cells may be better adapted to long-term colonization of snow habitats potentially giving them a selective advantage within such dynamic environments, yet to fully elucidate the underpinning mechanisms behind these advantages, more in depth and time-resolved studies in the field are necessary.

### Community Composition

The green and red snow were also markedly different in their microbial community compositions (**Figure 2**). Green snow was dominated by the snow algae *Microglena* sp. (**Figure 2**; Supplementary Table S1), which is likely a polar sub-clade of *Chlamydomonas*. The genus *Microglena* has undergone recent revisions (Demchenko et al., 2012) and shows strong adaptations to low temperatures (Leya, 2013). This species seems to thrive in high-nutrient environments with some shading from solar irradiation based on feedbacks with albedo and water content.

In contrast, the red snow from our sampling site was primarily represented through several *Chloromonas* species and two uncultured *Chlamydomonadaceae* species (**Figure 2**; Supplementary Table S1). Both the *Chloromonas* and *Chlamydomonadaceae* species are typical colonisers of red snow (Hoham and Duval, 2001; Leya et al., 2004; Remias et al., 2010b, 2013; Lutz et al., unpublished data). These two uncultured *Chlamydomonadaceae* show the highest sequence similarity with other *Chloromonas* species (Lutz et al., 2015). Despite being well adapted to harsh conditions, they were not abundant in green snow. One explanation could be the more mature life stage. This disfavors fast cell proliferation and may constitute a selective disadvantage compared to *Microglena*, which developed a high biomass in green snow and which may represent a species with a short life span without spore formation. Red snow algal species (*Chloromonas*) seem to be more oligotrophic K-strategists, whereas the green algae *Microglena* represents an r-strategist typical of nutrient rich environments.

In addition, the high algal biomass in both snow patches likely influenced the bacterial community. Although bacterial abundance was not quantified in our study, previous studies have found that bacterial numbers were on average one order of magnitude higher in red snow compared to snow with no visible algal communities colonizing them. Red snow has also been shown to have an at least ten times higher bacterial production rate suggesting a direct transfer of organic carbon from algae to bacteria, fixed through photosynthesis (Thomas and Duval, 1995). *Bacteriodetes* (e.g., *Flavobacterium*) and *Proteobacteria* (e.g., *Polaromonas, Rhodoferax, Janthinobacterium*), which were most abundant in both studied snow samples (**Figure 2**, Supplementary Table S2) are commonly found in Arctic snow (Harding et al., 2011; Møller et al., 2013), and are able to rapidly exploit organic matter (Riemann and Winding, 2001; Abell and Bowman, 2005). *Bacteriodetes* are known to be able to degrade complex organic structure and are often associated with environments of high organic content (Battin et al., 2001; Kirchman, 2002; Thomas et al., 2011). Among the *Bacteriodetes*, the *Flavobacteria* that were most abundant in green snow are often associated with freshwater phytoplankton blooms (Eiler and Bertilsson, 2004, 2007; Kolmonen et al., 2004). *Flavobacterium*, as well as *Saprospira*, that were most abundant in red snow, are also known to be microalgal pathogens (Afi et al., 1996; Salomon and Imai, 2006; Gachon et al., 2010), which may underpin a potential direct transfer of organic carbon from algae to bacteria.

The archaeal community in both samples was made up by *Nitrosophaeraceae* and *Cenarchaeaceae* (**Figure 2**; Supplementary Table S3), which are both known to be important ammonia-oxidizers (Tourna et al., 2011; Zarsky et al., 2013; Stieglmeier et al., 2014), but from our data no further conclusions could be drawn about them.

Although there were large differences in the algal and bacterial community composition between the green and red snow samples, the metagenomes of the two samples revealed few differences in main gene families (Supplementary Table S4; Supplementary Figure S1) implying that the same functions are covered by different species. However, small differences may not have been revealed due to the limited sequencing depth (Supplementary Table S6).

### Metabolic Profiles

Snow algal cells in the green and red snow analyzed in this study were not only different in terms of their community composition, but also in their metabolic profiles (**Table 4** and Supplementary Table S5, **Figure 3**). The green snow metabolic profile is more diverse than that of the red snow and is characterized by a more varied complement of metabolites. A large proportion of the green snow metabolites, which are likely predominantly derived from *Microglena*, could not be assigned putative identities. This is in accordance with Bundy et al. (2009), who reported that most environmental metabolic studies fail to identify a large proportion of metabolites. The variation in the metabolic profiles between the two snow samples also likely reflects the different stages in the respective life cycles at which the microbial communities inhabiting the green and red snow were sampled. It is noteworthy that *Microglena* has not been shown to inherit a resting spore stage, whereas this is the case for the *Chloromonas* species that were abundant in red snow (Hoham and Duval, 2001; Remias, 2012).

Nevertheless, metabolites found in the green snow, involved in the tryptophan degradation pathway (e.g., indole-3-pyruvate, IAA) were 10 times more abundant compared to red snow (**Table 4**; Supplementary Figure S3). The tryptophan degradation pathway results in the production of IAA, otherwise known as auxin. This well-characterized plant hormone has long been known to enhance growth of green algae such as *Chlorella pyrenoidosa* (Ahmad and Winter, 1968) and the high content of this metabolite supports the hypothesis that the algae in the green snow samples were in a growth stage of development. Alongside IAA, we also observed high concentrations of the pentose sugar ribose and lactose in our green snow samples (**Table 7**). Ribose is a monosaccharide that forms a constituent part of a variety of molecules involved in proliferation (e.g., ATP, RNA), while lactose is a disaccharide that is derived from galactose and glucose with the latter being synthesized during photosynthesis and so is again, indicative of growth and proliferation. Other metabolites that showed higher relative abundance in green snow were adenine and adenosine (mass numbers 135 and 267, **Table 4**), which are known to have an antioxidative, DNA-protective, and anti-inflammatory effect on nucleosides (da Rocha Lapa et al., 2012; Hartmann et al., 2015). Overall, the most discriminatory metabolites in the green snow sample suggest a general pattern of growth and proliferation, with intermediates of major biosynthetic pathways for key growth-modulating compounds being particularly abundant (**Table 4** and Supplementary Table S5, **Figure 3**).

In the red snow metabolic profiles, we found a high abundance of compounds putatively identified to be involved in purine metabolism (63%, **Table 4** and Supplementary Table S5), which is crucial in the synthesis of the nucleotides guanine and adenine (inosine monophosphate biosynthesis pathway; **Table 4**). Similarly, orotidine 5′-phosphate (Supplementary Figure S2), and sphinganine 1-phosphate were both also upregulated in red snow compared to green snow (**Table 4**). Sphinganine 1-phosphate is part of the sphingolipid metabolism, which synthesizes sphingolipids that are believed to inherit a variety of functions including hardening of cell surfaces to protect cells from the harmful environment (Sperling and Heinz, 2003). The upregulation of orotidine 5′-phosphate, which is part of the uridine monophosphate biosynthesis pathway (pyrimidine metabolism) may suggest that red snow algal spores were synthesizing ribonucleotides, possibly as storage compounds for overwintering. In this way, there would be a ready source of nitrogen for remobilization (Werner and Witte, 2011) in the spring to facilitate algal proliferation. There are other potential explanations for these observations, such as exposure to RNA-degrading UV radiation or another extreme abiotic condition, however, these do not account for the extreme difference in metabolic strategies between red and green snow samples given their close proximity within the field. It may be that the synthesis of ribonucleotides is associated with increased protein synthesis cells which may also facilitate algal overwintering.

Overall, for psychrotrophic bacteria it has been suggested that a rapid protein turnover, involving upregulation of RNA synthesis and therefore UMP compounds, and the mobilization/degradation of amino acids during synthesis of new proteins could be an energy-saving mechanism, particularly in low-nutrient environments like the red snow setting (Margesin and Schinner, 1994). Since red snow algal spores do not proliferate during this stage of their life cycle (Remias et al., 2013), energy can be invested in the accumulation of reserve metabolites to face impending severe conditions. This may involve becoming stranded on bare rock or ice after snow melt, which may be associated with higher temperatures, or desiccation stress or burial in deeper snow over winter with freezing and desiccation stress.

The red snow algae cells are nonetheless still photosynthetically active (Thomas and Duval, 1995), and thus they still require protection of their photosystem. This is mainly achieved through the secondary carotenoid synthesis, which made up 92% of the cells pigment content (as opposed to 3% in green snow, **Table 5**). In the red snow sample, the dominant secondary carotenoid astaxanthin was mainly mono- but also di-esterified with fatty acids, thus more lipophilic likely allowing membrane functioning at low temperatures (Remias et al., 2010b). In contrast, in green snow the pigments were mainly chlorophylls and primary carotenoids including violaxanthin, antheraxanthin, and zexanthin. These are all part of the xanthophyll cycle, which deviates excessive irradiation via removal of epoxy groups (Demmig-Adams and Adams, 1996). During light stress violaxanthin is converted into antheraxanthin and zeaxanthin (Goss and Jakob, 2010). The higher amount of the epoxidised xanthophyll violaxanthin compared to antheraxanthin and zeaxanthin in the green snow sample suggests relatively lower light stress levels. This could be because of the protective liquid water layer (as discussed above), a rather recent development of this snow algal bloom and hence less exposure to solar irradiation, or the deeper penetration of the snow and therefore a shading effect. In contrast, the drier red snow contained negligible amounts (4%) of primary carotenoids, and the high content of the secondary carotenoid astaxanthin (**Table 5**) indicates a different light stress response (Lemoine and Schoefs, 2010).

The link between the development of pigments and fatty acids is exemplified in the fact that the PUFA were most abundant in red snow, in particular C16:3, C16:4, C18:2, and C18:3, whereas in green snow they were absent (**Table 6**). This matches with the findings of Spijkerman et al. (2012) who reported the same fatty acid compounds in red snow algae from western Svalbard and Řezanka et al. (2008) who found that PUFAs accounted for 75% of total fatty acids in *Chloromonas brevisipina* collected from a snow field in the Czech Republic. Fatty acid composition can be affected by temperature, nutrient concentrations and solar radiation (Piorreck et al., 1984). We have recently shown a positive correlation for the abundance of PUFAs, secondary carotenoids and nutrient limitation in red snow algal spores (Lutz et al., unpublished data) and this is also well known for other green algae like *Haematococcus pluvialis* when exposed to light or nutrient stress (Lemoine and Schoefs, 2010). The high nutrient availability and the water film that led to lower light stress in the green snow in this study are likely the reason for the absence of PUFA but a high abundance of SFA.

In contrast, nutrients were limited in red snow and the low dissolved nitrate and phosphate concentrations were also reflected in the high particulate C/N and C/P ratios (**Table 3**). Under nitrogen and phosphorus limiting conditions, the metabolism is directed to N and P free metabolites such as lipids and carbohydrates. These were higher in red snow compared to green snow which contained a higher amount of proteins (**Table 8**). As already mentioned above, lipids are linked to carotenoids and specially astaxanthin since lipids can serve as storage molecules for lipophilic carotenoids during overwintering (Leya, 2013).

These bulk analyses have been verified also at the single cell level through the synchrotron radiation infrared analysis. In single green cells we confirmed the higher relative abundance of protein functional groups compared to the larger abundance of lipid and ester functional groups in all single red cells (**Tables 9A,B**). The esters in the red cells resulted from astaxanthin being esterified with the fatty acids.

The application of multi ‘omic’ approaches, and the combination of targeted vs. non-targeted and bulk vs. single cell analyses are in still in their infancy when the samples are derived from extreme environments. Specifically, the use of metabolomics in an environmental context is a relatively new technique that produces a huge amount of data. As such, results stemming from this type of analysis need to be interpreted with caution. However, an advantage of metabolomics is that it can be applied to all species without the knowledge of their genomes (Bundy et al., 2009) and certain metabolites may be very common among plants and algae. Here we have shown that, in combination with targeted metabolic studies, metabolomics is a powerful ecological tool and that the combination of such data with our results from other analyses allows us to be more confident in our data interpretation. Most metabolomics initiatives in recent years have focused on mammalian and in particular human metabolomes (Bundy et al., 2009), therefore more reference organisms need to be studied, under controlled laboratory conditions as well as in the field, and the identification of metabolites needs to be moved forward by targeted metabolic profiling of individual metabolic groups to unravel their functional roles.

Nevertheless, in the first comprehensive study of its kind, we showed that in an extreme natural environment where green and red snow clearly vary in their physico-chemical characteristics, their community composition and their metabolic profiles, these undeniably also reflect different stages of microbial life cycles and adaptation strategies. Our data suggests that green snow and red snow are not successive stages but two independent phenomena with different requirements to their environments. A variety of feedbacks exist between the algal communities and the physico-chemical environment they live in, including liquid water content, pH, albedo, and nutrient availability. Those feedbacks are most likely bidirectional, the environment affects algal distribution and function, but the algae also actively alter their environment. The differences in metabolic profiles are explained through growth and proliferation of the algae in the green snow, whereas accumulation and storage of reserve metabolites for upcoming severe conditions in the algae making up the red snow. It is, however, only through the power of combining a variety of established and new analytical approaches that we could ultimately elucidate a rather enigmatic environmental phenomena such as green and red snow algal blooms, which have an increased presence and crucial role in the fast melting polar and alpine glacial ecosystems.

## Author Contributions

SL and LB designed the study. Field work was carried out by SL, AA, and LB. All analyses were completed by SL. Metabolomics work was carried out by SL at the University of Sheffield under supervision of KF. All authors contributed to the discussion of the results. Manuscript was written by SL with inputs from AA, KF, and LB.

## Conflict of Interest Statement

The authors declare that the research was conducted in the absence of any commercial or financial relationships that could be construed as a potential conflict of interest.

## Footnotes

1 http://biocyc.org/

2 http://www.genome.jp/kegg/

## References

[B1] Abell G. Bowman J. P. (2005). Colonization and community dynamics of class Flavobacteria on diatom detritus in experimental mesocosms based on Southern Ocean seawater. *FEMS Microbiol. Ecol.* 53 379–391. 10.1016/j.femsec.2005.01.008 16329957

[B2] Afi L. Metzger P. Largeau C. Connan J. Berkaloff C. Rousseau B. (1996). Bacterial degradation of green microalgae: incubation of *Chlorella emersonii* and *Chlorella vulgaris* with *Pseudomonas oleovorans* and *Flavobacterium aquatile*. *Org. Geochem.* 25 117–130. 10.1016/S0146-6380(96)00113-1

[B3] Agati G. Azzarello E. Pollastri S. Tattini M. (2012). Flavonoids as antioxidants in plants: location and functional significance. *Plant Sci.* 196 67–76. 10.1016/j.plantsci.2012.07.014 23017900

[B4] Ahmad M. R. Winter A. (1968). Studies on the hormonal relationships of algae in pure culture. *Planta* 81 16–27. 10.1007/BF00385511 24519593

[B5] Battin T. J. Wille A. Sattler B. Psenner R. (2001). Phylogenetic and functional heterogeneity of sediment biofilms along environmental gradients in a glacial stream. *Appl. Environ. Microbiol.* 67 799–807. 10.1128/AEM.67.2.799-807.2001 11157246 PMC92650

[B6] Benning L. G. Anesio A. M. Lutz S. Tranter M. (2014). Biological impact on Greenland’s albedo. *Nat. Geosci.* 7 691–691. 10.1038/ngeo2260

[B7] Benning L. G. Phoenix V. Yee N. Tobin M. (2004). Molecular characterization of cyanobacterial silicification using synchrotron infrared micro-spectroscopy. *Geochim. Cosmochim. Acta* 68 729–741. 10.1016/S0016-7037(03)00489-7

[B8] Bidigare R. Ondrusek M. Kennicutt M. Iturriaga R. Harvey H. Hoham R. (1993). Evidence for a photoprotective function for secondary carotenoids of snow algae. *J. Phycol.* 29 427–434. 10.1111/j.1529-8817.1993.tb00143.x

[B9] Bundy J. G. Davey M. P. Viant M. R. (2009). Environmental metabolomics: a critical review and future perspectives. *Metabolomics* 5 3–21. 10.1007/s11306-008-0152-0

[B10] Caporaso J. G. Kuczynski J. Stombaugh J. Bittinger K. Bushman F. D. Costello E. K. (2010). QIIME allows analysis of high-throughput community sequencing data. *Nat. Methods* 7 335–336. 10.1038/nmeth.f.303 20383131 PMC3156573

[B11] Cheung M. K. Au C. H. Chu K. H. Kwan H. S. Wong C. K. (2010). Composition and genetic diversity of picoeukaryotes in subtropical coastal waters as revealed by 454 pyrosequencing. *ISME J.* 4 1053–1059. 10.1038/ismej.2010.26 20336159

[B12] Czygan F.-C. (1970). Blutregen und blutschnee: stickstoffmangel-zellen von *Haematococcus pluvialis* und *Chlamydomonas nivalis*. *Archiv. Mikrobiol.* 74 69–76. 10.1007/BF00408689 5486496

[B13] da Rocha Lapa F. Da Silva M. D. De Almeida Cabrini D. Santos A. R. (2012). Anti-inflammatory effects of purine nucleosides, adenosine and inosine, in a mouse model of pleurisy: evidence for the role of adenosine A2 receptors. *Purinerg. Signal.* 8 693–704. 10.1007/s11302-012-9299-2 PMC3486164 22456813

[B14] Demchenko E. Mikhailyuk T. Coleman A. W. Pröschold T. (2012). Generic and species concepts in Microglena (previously the *Chlamydomonas monadina* group) revised using an integrative approach. *Eur. J. Phycol.* 47 264–290. 10.1080/09670262.2012.678388

[B15] Demmig-Adams B. Adams W. W. (1996). The role of xanthophyll cycle carotenoids in the protection of photosynthesis. *Trends Plant Sci.* 1 21–26. 10.1016/S1360-1385(96)80019-7

[B16] DeSantis T. Z. Hugenholtz P. Larsen N. Rojas M. Brodie E. L. Keller K. (2006). Greengenes, a chimera-checked 16S rRNA gene database and workbench compatible with ARB. *Appl. Environ. Microbiol.* 72 5069–5072. 10.1128/AEM.03006-05 16820507 PMC1489311

[B17] Dineley D. (1958). A review of the Carboniferous and Permian rocks of the west coast of Vestspitsbergen. *Nors. Geolog. Tidsskr.* 38 197–217.

[B18] Duval B. Shetty K. Thomas W. H. (1999). Phenolic compounds and antioxidant properties in the snow alga *Chlamydomonas nivalis* after exposure to UV light. *J. Appl. Phycol.* 11 559–566. 10.1023/A:1008178208949

[B19] Eiler A. Bertilsson S. (2004). Composition of freshwater bacterial communities associated with cyanobacterial blooms in four Swedish lakes. *Environ. Microbiol.* 6 1228–1243. 10.1111/j.1462-2920.2004.00657.x 15560821

[B20] Eiler A. Bertilsson S. (2007). Flavobacteria blooms in four eutrophic lakes: linking population dynamics of freshwater bacterioplankton to resource availability. *Appl. Environ. Microbiol.* 73 3511–3518. 10.1128/AEM.02534-06 17435002 PMC1932693

[B21] Field K. J. Lake J. A. (2011). Environmental metabolomics links genotype to phenotype and predicts genotype abundance in wild plant populations. *Physiol. Plant* 142 352–360. 10.1111/j.1399-3054.2011.01480.x 21496032

[B22] Fogg G. (1967). Observations on the snow algae of the South Orkney Islands. *Philos. Trans. R. Soc. Lond. B Biol. Sci.* 252 279–287. 10.1098/rstb.1967.0018

[B23] Fujii M. Takano Y. Kojima H. Hoshino T. Tanaka R. Fukui M. (2010). Microbial community structure, pigment composition, and nitrogen source of red snow in Antarctica. *Microb. Ecol.* 59 466–475. 10.1007/s00248-009-9594-9 19847476 PMC4261141

[B24] Gachon C. M. Sime-Ngando T. Strittmatter M. Chambouvet A. Kim G. H. (2010). Algal diseases: spotlight on a black box. *Trends Plant Sci.* 15 633–640. 10.1016/j.tplants.2010.08.005 20833575

[B25] Gentz-Werner P. (2007). *Roter Schnee: oder Die Suche nach dem färbenden Prinzip.* Berlin: Akademie Verlag.

[B26] Goss R. Jakob T. (2010). Regulation and function of xanthophyll cycle-dependent photoprotection in algae. *Photosynthesis Res.* 106 103–122. 10.1007/s11120-010-9536-x 20224940

[B27] Häder D.-P. Häder M. A. (1989). Effects of solar UV-B irradiation on photomovement and motility in photosynthetic and colorless flagellates. *Environ. Exp. Bot.* 29 273–282. 10.1016/0098-8472(89)90059-2

[B28] Harding T. Jungblut A. D. Lovejoy C. Vincent W. F. (2011). Microbes in high Arctic snow and implications for the cold biosphere. *Appl. Environ. Microbiol.* 77 3234–3243. 10.1128/AEM.02611-10 21460114 PMC3126466

[B29] Harland W. (1997). *The Geology of Svalbard. Geological Society Memoir* 17 London: The Geological Society.

[B30] Hartmann A. Albert A. Ganzera M. (2015). Effects of elevated ultraviolet radiation on primary metabolites in selected alpine algae and cyanobacteria. *J. Photochem. Photobiol. B Biol.* 149 149–155. 10.1016/j.jphotobiol.2015.05.016 PMC4509709 26065817

[B31] Hoham R. Duval B. (2001). *Microbial Ecology of Snow and Freshwater Ice with Emphasis on Snow Algae.* Cambridge: Cambridge University Press.

[B32] Jamers A. Blust R. De Coen W. (2009). Omics in algae: paving the way for a systems biological understanding of algal stress phenomena? *Aquat. Toxicol.* 92 114–121. 10.1016/j.aquatox.2009.02.012 19304329

[B33] Kent W. J. (2002). BLAT—the BLAST-like alignment tool. *Genome Res.* 12 656–664. 10.1101/gr.229202 11932250 PMC187518

[B34] Kirchman D. L. (2002). The ecology of *Cytophaga*–Flavobacteria in aquatic environments. *FEMS Microbiol. Ecol.* 39 91–100. 10.1111/j.1574-6941.2002.tb00910.x 19709188

[B35] Kol E. (1968). *Kryobiologie; Biologie und Limnologie des Schnees und Eises.* Stuttgart: Schweizerbart’sche Verlagsbuchhandlung.

[B36] Kolmonen E. Sivonen K. Rapala J. Haukka K. (2004). Diversity of cyanobacteria and heterotrophic bacteria in cyanobacterial blooms in Lake Joutikas, Finland. *Aquat. Microbiol. Ecol.* 36 201–211. 10.3354/ame036201

[B37] Kutchan T. Dixon R. A. (2005). Physiology and metabolism: secondary metabolism: nature’s chemical reservoir under deconvolution. *Curr. Opin. Plant Biol.* 8 227–229. 10.1016/j.pbi.2005.03.016

[B38] Lemoine Y. Schoefs B. (2010). Secondary ketocarotenoid astaxanthin biosynthesis in algae: a multifunctional response to stress. *Photosynth. Res.* 106 155–177. 10.1007/s11120-010-9583-3 20706789

[B39] Leya T. (2013). “Snow algae: adaptation strategies to survive on snow and ice,” in *Polyextremophiles*, eds Seckbach J. Oren A. Stan-Lotter H. (Berlin: Springer), 401–423.

[B40] Leya T. Müller T. Ling H. U. Fuhr G. (2004). “Snow algae from north-western Spitsbergen (Svalbard),” in *The Coastal Ecosystem of Kongsfjorden, Svalbard. Synopsis of Biological Research Performed at the Koldewey Station in the Years 1991-2003* Vol. 492 ed. Wiencke C. (Bremerhaven: Alfred Wegener Institute for Polar and Marine Research), 46–54.

[B41] Leya T. Rahn A. Lütz C. Remias D. (2009). Response of arctic snow and permafrost algae to high light and nitrogen stress by changes in pigment composition and applied aspects for biotechnology. *FEMS Microbiol. Ecol.* 67 432–443. 10.1111/j.1574-6941.2008.00641.x 19159422

[B42] Lutz S. Anesio A. M. Edwards A. Benning L. G. (2015). Microbial diversity on Icelandic glaciers and ice caps. *Front. Microbiol.* 6:307 10.3389/fmicb.2015.00307 PMC4403510 25941518

[B43] Lutz S. Anesio A. M. Villar S. E. J. Benning L. G. (2014). Variations of algal communities cause darkening of a Greenland glacier. *FEMS Microbiol. Ecol.* 89 402–414. 10.1111/1574-6941.12351 24920320

[B44] Margesin R. Schinner F. (1994). Properties of cold-adapted microorganisms and their potential role in biotechnology. *J. Biotechnol.* 33 1–14. 10.1016/0168-1656(94)90093-0

[B45] Matsuzaki R. Kawai-Toyooka H. Hara Y. Nozaki H. (2015). Revisiting the taxonomic significance of aplanozygote morphologies of two cosmopolitan snow species of the genus Chloromonas (Volvocales, Chlorophyceae). *Phycologia* 54 491–502. 10.2216/15-33.1

[B46] Merchant S. S. Prochnik S. E. Vallon O. Harris E. H. Karpowicz S. J. Witman G. B. (2007). The *Chlamydomonas* genome reveals the evolution of key animal and plant functions. *Science* 318 245–250. 10.1126/science.1143609 17932292 PMC2875087

[B47] Møller A. K. Søborg D. A. Al-Soud W. A. Sørensen S. J. Kroer N. (2013). Bacterial community structure in High-Arctic snow and freshwater as revealed by pyrosequencing of 16S rRNA genes and cultivation. *Polar Res.* 32:17390 10.3402/polar.v32i0.17390

[B48] Mueller D. R. Vincent W. F. Pollard W. H. Fritsen C. H. (2001). Glacial cryoconite ecosystems: a bipolar comparison of algal communities and habitats. *Nova Hedwigia Beiheft* 123 173–198.

[B49] Overy S. Walker H. Malone S. Howard T. Baxter C. Sweetlove L. (2005). Application of metabolite profiling to the identification of traits in a population of tomato introgression lines. *J. Exp. Bot.* 56 287–296. 10.1093/jxb/eri070 15596481

[B50] Piorreck M. Baasch K.-H. Pohl P. (1984). Biomass production, total protein, chlorophylls, lipids and fatty acids of freshwater green and blue-green algae under different nitrogen regimes. *Phytochemistry* 23 207–216. 10.1016/S0031-9422(00)80305-2

[B51] Ramel F. Birtic S. Cuiné S. Triantaphylidès C. Ravanat J.-L. Havaux M. (2012). Chemical quenching of singlet oxygen by carotenoids in plants. *Plant Physiol.* 158 1267–1278. 10.1104/pp.111.182394 22234998 PMC3291260

[B52] Remias D. (2012). *Cell Structure and Physiology of Alpine Snow and Ice Algae.* Vienna: Springer.

[B53] Remias D. Albert A. Lütz C. (2010a). Effects of realistically simulated, elevated UV irradiation on photosynthesis and pigment composition of the alpine snow alga *Chlamydomonas nivalis* and the arctic soil alga Tetracystis sp.(Chlorophyceae). *Photosynthetica* 48 269–277. 10.1007/s11099-010-0033-4

[B54] Remias D. Karsten U. Lütz C. Leya T. (2010b). Physiological and morphological processes in the Alpine snow alga *Chloromonas nivalis* (Chlorophyceae) during cyst formation. *Protoplasma* 243 73–86. 10.1007/s00709-010-0123-y 20229328

[B55] Remias D. Lutz C. (2007). Characterisation of esterified secondary carotenoids and of their isomers in green algae: a HPLC approach. *Algolog. Stud.* 124 85–94. 10.1127/1864-1318/2007/0124-0085

[B56] Remias D. Lütz-Meindl U. Lütz C. (2005). Photosynthesis, pigments and ultrastructure of the alpine snow alga *Chlamydomonas nivalis*. *Eur. J. Phycol.* 40 259–268. 10.1080/09670260500202148

[B57] Remias D. Wastian H. Lütz C. Leya T. (2013). Insights into the biology and phylogeny of Chloromonas polyptera (Chlorophyta), an alga causing orange snow in Maritime Antarctica. *Antarc. Sci.* 25 1–9.

[B58] Řezanka T. Nedbalová L. Sigler K. (2008). Unusual medium-chain polyunsaturated fatty acids from the snow alga *Chloromonas brevispina*. *Microbiol. Res.* 163 373–379. 10.1016/j.micres.2006.11.021 17403601

[B59] Riemann L. Winding A. (2001). Community dynamics of free-living and particle-associated bacterial assemblages during a freshwater phytoplankton bloom. *Microb. Ecol.* 42 274–285. 10.1007/s00248-001-0018-8 12024253

[B60] Ruttenberg K. Ogawa N. Tamburini F. Briggs R. Colasacco N. Joyce E. (2009). Improved, high-throughput approach for phosphorus speciation in natural sediments via the SEDEX sequential extraction method. *Limnol. Oceanogr. Methods* 7 319–333. 10.4319/lom.2009.7.319

[B61] Salomon P. Imai I. (2006). “Pathogens of harmful microalgae,” in *Ecology of Harmful Algae*, eds Granéli E. Turner J. T. (Berlin: Springer), 271–282.

[B62] Sperling P. Heinz E. (2003). Plant sphingolipids: structural diversity, biosynthesis, first genes and functions. *Biochim. Biophys. Acta Mol. Cell Biol. Lipids* 1632 1–15. 10.1016/S1388-1981(03)00033-7 12782146

[B63] Spijkerman E. Wacker A. Weithoff G. Leya T. (2012). Elemental and fatty acid composition of snow algae in Arctic habitats. *Front. Microbiol.* 3:380 10.3389/fmicb.2012.00380 PMC3482990 23112797

[B64] Stibal M. Elster J. Šabacká M. Kaštovská K. (2007). Seasonal and diel changes in photosynthetic activity of the snow alga *Chlamydomonas nivalis* (Chlorophyceae) from Svalbard determined by pulse amplitude modulation fluorometry. *FEMS Microbiol. Ecol.* 59 265–273. 10.1111/j.1574-6941.2006.00264.x 17313577

[B65] Stieglmeier M. Klingl A. Alves R. J. Simon K.-M. R. Melcher M. Leisch N. (2014). *Nitrososphaera viennensis* sp. nov., an aerobic and mesophilic ammonia-oxidizing archaeon from soil and member of the archaeal phylum Thaumarchaeota. *Int. J. Syst. Evol. Microbiol.* 64 2738–2752. 10.1099/ijs.0.063172-0 24907263 PMC4129164

[B66] Takeuchi N. (2002). The altitudinal distribution of snow algae on an Alaska glacier (Gulkana Glacier in the Alaska Range). *Hydrolog. Process.* 15 3447–3459. 10.1002/hyp.1040

[B67] Thomas F. Hehemann J.-H. Rebuffet E. Czjzek M. Michel G. (2011). Environmental and gut bacteroidetes: the food connection. *Front. Microbiol.* 2:93 10.3389/fmicb.2011.00093 PMC3129010 21747801

[B68] Thomas W. H. Duval B. (1995). Sierra Nevada, California, USA, snow algae: snow albedo changes, algal-bacterial interrelationships, and ultraviolet radiation effects. *Arct. Alp. Res.* 27 389–399. 10.2307/1552032

[B69] Tourna M. Stieglmeier M. Spang A. Könneke M. Schintlmeister A. Urich T. (2011). *Nitrososphaera viennensis*, an ammonia oxidizing archaeon from soil. *Proc. Natl. Acad. Sci. U.S.A.* 108 8420–8425. 10.1073/pnas.1013488108 21525411 PMC3100973

[B70] Viant M. R. (2007). Metabolomics of aquatic organisms: the new ‘omics’ on the block. *Marine Ecol. Prog. Series* 332 301–306. 10.3354/meps332301

[B71] Vickers C. E. Gershenzon J. Lerdau M. T. Loreto F. (2009). A unified mechanism of action for volatile isoprenoids in plant abiotic stress. *Nat. Chem. Biol.* 5 283–291. 10.1038/nchembio.158 19377454

[B72] Wacker A. Martin-Creuzburg D. (2007). Allocation of essential lipids in *Daphnia magna* during exposure to poor food quality. *Funct. Ecol.* 21 738–747. 10.1111/j.1365-2435.2007.01274.x

[B73] Werner A. K. Witte C.-P. (2011). The biochemistry of nitrogen mobilization: purine ring catabolism. *Trends Plant Sci.* 16 381–387. 10.1016/j.tplants.2011.03.012 21482173

[B74] Yallop M. L. Anesio A. M. Perkins R. G. Cook J. Telling J. Fagan D. (2012). Photophysiology and albedo-changing potential of the ice algal community on the surface of the Greenland ice sheet. *ISME J.* 6 2302–2313. 10.1038/ismej.2012.107 23018772 PMC3504962

[B75] Yoshimura Y. Kohshima S. Takeuchi N. Seko K. Fujita K. (2006). Snow algae in a Himalayan ice core: new environmental markers for ice-core analyses and their correlation with summer mass balance. *Ann. Glaciol.* 43 148–153. 10.3189/172756406781812276

[B76] Zarsky J. D. Stibal M. Hodson A. Sattler B. Schostag M. Hansen L. H. (2013). Large cryoconite aggregates on a Svalbard glacier support a diverse microbial community including ammonia-oxidizing archaea. *Environ. Res. Lett.* 8 035044 10.1088/1748-9326/8/3/035044

