# Supplementary material for: Integrated ‘Omics’, Targeted Metabolite and Single-cell Analyses of Arctic Snow Algae Functionality and Adaptability
Source: Front Microbiol. 2015 Nov 25;6:1323. doi: 10.3389/fmicb.2015.01323 (PMC4659291; doi:10.3389/fmicb.2015.01323)
Supplement: Supplementary file 1 [file Data_Sheet_1.DOCX]

Supplementary Material

**Integrated ‘omics’, targeted metabolite and single-cell analyses of Arctic snow algae functionality and adaptability**

**Stefanie Lutz^1,2*^, Alexandre M. Anesio^3^, Katie Field^4^**^§^**, Liane G. Benning^1,2*^**

^1^Cohen Laboratories, School of Earth & Environment, University of Leeds, Leeds, LS2 9JT, UK

^2^GFZ German Research Centre for Geosciences, Telegrafenberg, 14473 Potsdam, Germany

^3^Bristol Glaciology Centre, School of Geographical Sciences, University of Bristol, Bristol, BS8 1SS, UK

^4^Department of Animal and Plant Sciences, University of Sheffield, Western Bank, Sheffield, S10 2TN

^§^Present address: School of Biology, Faculty of Biological Sciences, University of Leeds, Leeds, LS2 9JT, UK

*** Correspondence:** Stefanie Lutz (s.lutz@leeds.ac.uk) or Liane G. Benning (benning@gfz-potsdam.de)

# Supplementary Figures and Tables

Pages: 11

Figures: 3

Tables: 6

## Supplementary Figures

Figure S1: Metagenomes showing proportions of main gene families in green and red snow. No major differences at this sequencing depth.


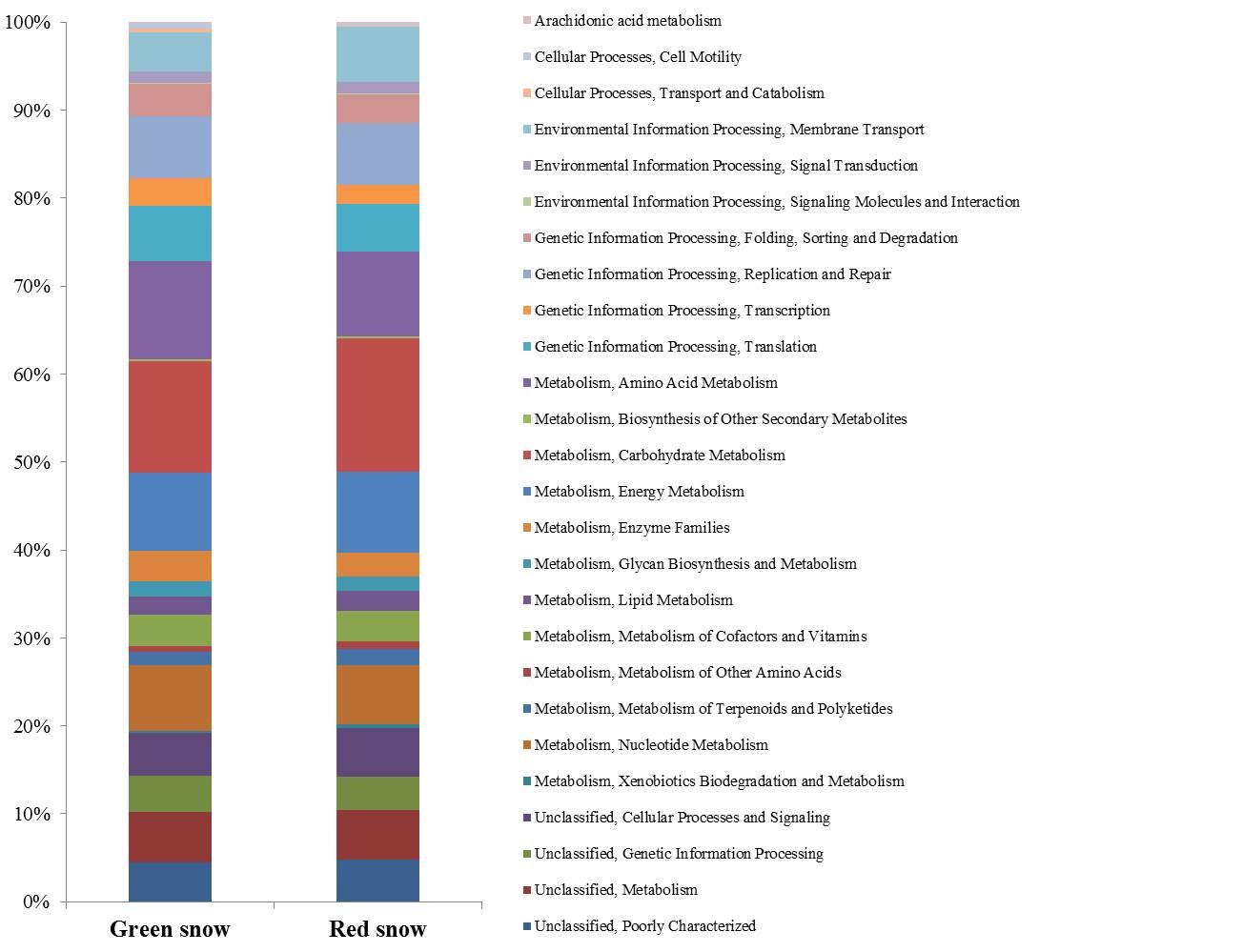


**Figure S2**: Metabolomics data mapped on uridine monophosphate (UMP) biosynthesis pathway. Numbers represent relative abundance (in %) of metabolites for green (green boxes) and red (red boxes) snow.


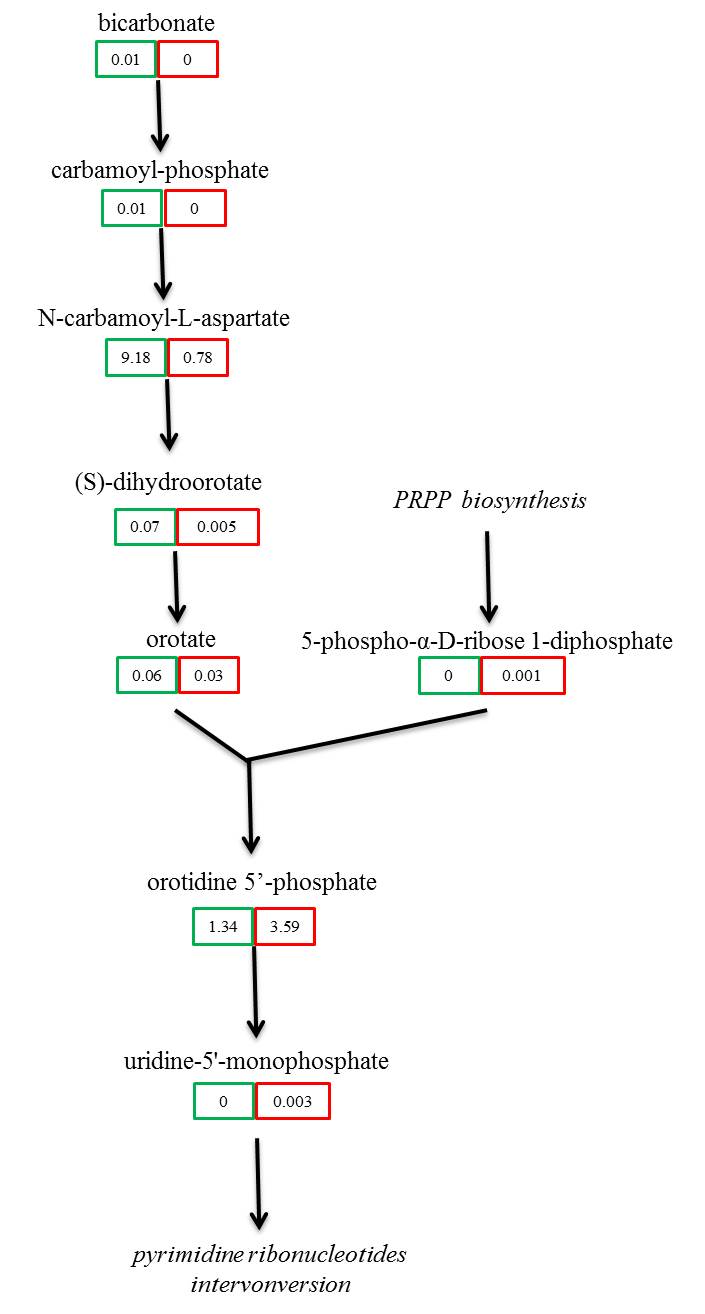


Figure S3: Metabolomics data mapped on tryptophan degradation pathway. Numbers represent relative abundance (in %) of metabolites for green (green boxes) and red (red boxes) snow.


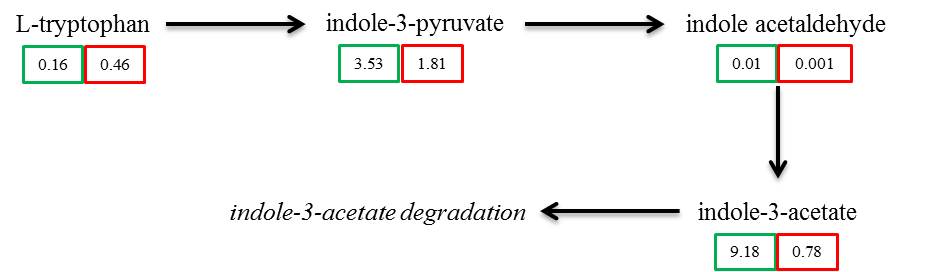


## Supplementary Tables

**Table S1:** Distribution of 97% clustered OTUs aligned and assigned to *Archaeplastida* (green algae). Values are the relative abundance of the taxa in percentage of total sequences and table shows taxa with OTUs of a minimum total observation count of 0.1%.

|  | Green snow | Red snow |
| --- | --- | --- |
| *Chlamydomonas cf. proboscigera* CCCryo 217-05 | 0.57 | 0.00 |
| *Chloromonas cf. alpina* CCCryo 033-99 (AF514403) | 0.09 | 5.02 |
| *Chloromonas cf. platystigma* CCCryo 020-99 | 0.03 | 1.64 |
| *Chloromonas nivalis (*AF514409) | 0.24 | 48.24 |
| *Chloromonas polyptera (*JQ790556) | 0.06 | 13.13 |
| *Chloromonas tughillensis* | 0.06 | 1.50 |
| *Microglena sp.* (polar subclade *Chlamydomonas*) (EF537906*)* | 98.52 | 0.09 |
| *Raphidonema sempervirens* (AJ309939) | 0.33 | 3.61 |
| Uncultured *Chlamydomonadaceae* (1) | 0.00 | 3.00 |
| Uncultured *Chlamydomonadaceae* (2) (GU117577) | 0.09 | 23.68 |

**Table S2:** Distribution of 97% clustered OTUs aligned and assigned to known bacterial classes (bold) and the orders, families and genera therein (indented), where possible (“__” = lack of assignment depth). Values are the relative abundance of the taxa in percentage of total sequences and taxa with >0.01% abundance are shown.

| Taxon | Green snow | Red snow |
| --- | --- | --- |
| *Actinobacteria, Actinobacteria* | 0.08 | 1.03 |
| *Actinomycetales; Sporichthyaceae; __* | 0.08 | 1.03 |
| *Bacteriodetes, Cytophagia* | 3.61 | 0.88 |
| *Cytophagales, Cytophagaceae, Flectobacillus* | 3.61 | 0.88 |
|  |  |  |
| *Bacteriodetes, Flavobacteria* | 76.84 | 0.00 |
| *Flavobacteriales, Flavobacteriaceae, Flavobacterium* | 76.84 | 0.00 |
|  |  |  |
| *Bacteriodetes, Saprospirae* | 2.80 | 86.93 |
| *Saprospirales, Chitinophagaceae, __* | 2.80 | 86.93 |
|  |  |  |
| *Chlorobi, Ignavibacteria* | 2.00 | 1.62 |
| *Ignavibacteriales, Ignavibacteriaceae, __* | 2.00 | 1.62 |
|  |  |  |
| *Proteobacteria, Alphaproteobacteria* | 4.25 | 0.37 |
| *__, __, __* | 2.88 | 0.37 |
| *BD7-3, __, __* | 0.96 | 0.00 |
| *Rickettsiales, Pelagibacteraceae, __* | 0.40 | 0.00 |
|  |  |  |
| *Proteobacteria, Betaproteobacteria* | 10.42 | 9.18 |
| *Burkholderiales, __, __* | 0.16 | 2.86 |
| *Burkholderiales, Comamonadaceae, __* | 1.60 | 0.00 |
| *Burkholderiales, Comamonadaceae, Methylibium* | 0.32 | 0.81 |
| *Burkholderiales, Comamonadaceae, Paucibacter* | 0.16 | 1.47 |
| *Burkholderiales, Comamonadaceae, Polaromonas* | 0.00 | 0.44 |
| *Burkholderiales, Comamonadaceae, Rhodoferax* | 0.32 | 2.42 |
| *Burkholderiales, Comamonadaceae, Rubrivivax* | 0.40 | 0.00 |
| *Burkholderiales, Oxalobacteraceae, __* | 0.80 | 0.00 |
| *Burkholderiales, Oxalobacteraceae, Janthinobacterium* | 0.40 | 0.00 |
| *Gallionellales, Gallionellaceae, Gallionella* | 5.53 | 1.10 |
| *Rhodocyclales, Rhodocyclaceae, __* | 0.72 | 0.07 |

Table S3: Distribution of 97% clustered OTUs aligned and assigned to archaea.

| Taxon | Green snow | Red snow |
| --- | --- | --- |
| *Thaumarchaeota, Cenarchaeaceae* | 0.03 | 25.63 |
| *Thaumarchaeota, Nitrososphaeraceae* | 99.93 | 74.34 |

Table S4: Relative abundance of functional categories for the annotated genes in the metagenomes of green and red snow with relevant sub-categories (underlined) and KEGG orthology (KO) identifiers. Gene categories that were also represented in the metabolomes are in bold (see also Table 4).

| Level 1, Level 2 | Level 3 | Level 4 | Green snow [%] | Red snow [%] | KO |
| --- | --- | --- | --- | --- | --- |
| Cellular Processes, Cell Motility |  |  | 0.61 | 0.18 |  |
| Cellular Processes, Transport and Catabolism |  |  | 0.41 | 0.16 |  |
| Environmental Information Processing, Membrane Transport |  |  | 4.44 | 6.26 |  |
| Environmental Information Processing, Signal Transduction |  |  | 1.26 | 1.25 |  |
| Environmental Information Processing, Signaling Molecules and Interaction |  |  | 0.18 | 0.08 |  |
| Genetic Information Processing, Folding, Sorting and Degradation |  |  | 3.68 | 3.33 |  |
| Genetic Information Processing, Replication and Repair |  |  | 7.01 | 7.03 |  |
| Genetic Information Processing, Transcription |  |  | 3.07 | 2.10 |  |
| Genetic Information Processing, Translation |  |  | 6.26 | 5.48 |  |
| Metabolism, Amino Acid Metabolism |  |  | 11.21 | 9.58 |  |
|  | Alanine, aspartate and glutamate metabolism |  | 0.43 | 0.42 |  |
|  | Amino acid related enzymes |  | 2.43 | 2.23 |  |
|  | Arginine and proline metabolism |  | 0.91 | 0.87 |  |
|  | Cysteine and methionine metabolism |  | 0.83 | 0.96 |  |
|  | Glycine, serine and threonine metabolism |  | 1.37 | 1.21 |  |
|  | Histidine metabolism |  | 0.85 | 0.48 |  |
|  | Lysine biosynthesis |  | 0.53 | 0.52 |  |
|  | Lysine degradation |  | 1.04 | 0.26 |  |
|  | Phenylalanine metabolism |  | 0.15 | 0.22 |  |
|  | Phenylalanine, tyrosine and tryptophan biosynthesis |  | 0.62 | 0.69 |  |
|  | **Tryptophan metabolism** |  | 0.28 | 0.16 |  |
|  |  | L-tryptophan aminotransferase | 0 | 0 | K16903 |
|  |  | indolepyruvate decarboxylase | 0 | 0 | [K04103](http://www.genome.jp/dbget-bin/www_bget?ko:K04103) |
|  |  | indole-3-acetaldehyde | 0 | 0 | [K11817](http://www.genome.jp/dbget-bin/www_bget?ko:K11817) |
|  | **Tyrosine metabolism** |  | 0.46 | 0.31 |  |
|  | Valine, leucine and isoleucine biosynthesis |  | 0.92 | 0.85 |  |
|  | Valine, leucine and isoleucine degradation |  | 0.40 | 0.40 |  |
| Metabolism, Biosynthesis of Other Secondary Metabolites |  |  | 0.19 | 0.24 |  |
|  | **Flavonoid biosynthesis** |  | 0.06 | 0.02 |  |
|  |  | 5-amino-6-(5-phospho-D-ribitylamino)  uracil phosphatase |  |  |  |
| Metabolism, Carbohydrate Metabolism |  |  | 12.68 | 15.15 |  |
|  | Amino sugar and nucleotide sugar metabolism |  | 0.82 | 1.14 |  |
|  | Ascorbate and aldarate metabolism |  | 0.04 | 0.08 |  |
|  | Butanoate metabolism |  | 0.85 | 0.97 |  |
|  | C5-Branched dibasic acid metabolism |  | 0.10 | 0.14 |  |
|  | Citrate cycle (TCA cycle) |  | 1.67 | 1.76 |  |
|  | **Fructose and mannose metabolism** |  | 0.80 | 1.00 |  |
|  |  | mannitol dehydrogenase | 0 | 0 | [K00045](http://www.genome.jp/dbget-bin/www_bget?ko:K00045) |
|  |  | mannitol-1-phosphatase |  |  |  |
|  |  | mannitol-1-phosphate dehydrogenase | 0 | 0 | [K00009](http://www.genome.jp/dbget-bin/www_bget?ko:K00009) |
|  |  | pyrophosphate-dependent  phosphofructokinase | 0 | 0 | [K00895](http://www.genome.jp/dbget-bin/www_bget?ko:K00895) |
|  |  | fructokinase | 0.02 | 0.01 | [K00847](http://www.genome.jp/dbget-bin/www_bget?ko:K00847) |
|  | Galactose metabolism |  | 0.60 | 0.69 |  |
|  | Glycolysis / Gluconeogenesis |  | 2.36 | 2.40 |  |
|  | Glyoxylate and dicarboxylate metabolism |  | 1.19 | 1.43 |  |
|  | Inositol phosphate metabolism |  | 0.21 | 0.11 |  |
|  | Pentose and glucuronate interconversions |  | 0.30 | 0.90 |  |
|  | Pentose phosphate pathway |  | 0.70 | 0.99 |  |
|  | Propanoate metabolism |  | 0.88 | 0.96 |  |
|  | Pyruvate metabolism |  | 1.21 | 1.51 |  |
|  | Starch and sucrose metabolism |  | 0.97 | 1.07 |  |
| Metabolism, Energy Metabolism |  |  | 8.85 | 9.23 |  |
|  | Carbon fixation in photosynthetic organisms |  | 0.21 | 0.18 |  |
|  | Carbon fixation pathways in prokaryotes |  | 0.16 | 0.23 |  |
|  | Methane metabolism |  | 0.70 | 1.02 |  |
|  | Nitrogen metabolism |  | 1.55 | 1.61 |  |
|  | Oxidative phosphorylation |  | 3.82 | 4.17 |  |
|  | Photosynthesis |  | 2.16 | 1.42 |  |
|  | Photosynthesis - antenna proteins |  | 0.01 | 0.00 |  |
|  | Photosynthesis proteins |  | 0.00 | 0.01 |  |
|  | Sulfur metabolism |  | 0.23 | 0.59 |  |
| Metabolism, Enzyme Families |  |  | 3.42 | 2.70 |  |
| Metabolism, Glycan Biosynthesis and Metabolism |  |  | 1.76 | 1.64 |  |
| Metabolism, Lipid Metabolism |  |  | 2.07 | 2.29 |  |
|  | Biosynthesis of unsaturated fatty acids |  | 0.16 | 0.11 |  |
|  | Fatty acid biosynthesis |  | 0.71 | 0.66 |  |
|  | Fatty acid metabolism |  | 0.30 | 0.32 |  |
|  | Glycerolipid metabolism |  | 0.20 | 0.17 |  |
|  | Glycerophospholipid metabolism |  | 0.33 | 0.39 |  |
|  | Linoleic acid metabolism |  | 0.07 | 0.05 |  |
|  | Lipid biosynthesis proteins |  | 0.07 | 0.13 |  |
|  | Primary bile acid biosynthesis |  | 0.00 | 0.01 |  |
|  | **Sphingolipid metabolism** |  | 0.04 | 0.18 |  |
|  | Steroid biosynthesis |  | 0.03 | 0.00 |  |
|  | Steroid hormone biosynthesis |  | 0.01 | 0.02 |  |
| Arachidonic acid metabolism |  |  | 0.14 | 0.24 |  |
| Metabolism, Metabolism of Cofactors and Vitamins |  |  | 3.47 | 3.44 |  |
|  | Riboflavin metabolism |  | 0.20 | 0.26 |  |
| Metabolism, Metabolism of Other Amino Acids |  |  | 0.75 | 0.84 |  |
| Metabolism, Metabolism of Terpenoids and Polyketides |  |  | 1.48 | 1.82 |  |
|  | Carotenoid biosynthesis |  | 0.14 | 0.09 |  |
|  |  | zeta-carotene desaturase | 0.0047 | 0.0000 | K00514 |
|  |  | 15-cis-phytoene desaturase | 0.0047 | 0.0000 | K02293 |
|  |  | beta-carotene hydroxylase | 0.0375 | 0.0057 | K02294 |
|  |  | lycopene beta-cyclase | 0.0141 | 0.0000 | K06443 |
|  |  | prolycopene isomerase | 0.0141 | 0.0000 | K09835 |
|  |  | phytoene desaturase | 0.0609 | 0.0738 | K10027 |
|  |  | diapolycopene oxygenase | 0.0000 | 0.0057 | K10210 |
| Metabolism, Nucleotide Metabolism |  |  | 7.43 | 6.76 |  |
|  | **Purine metabolism** |  | 5.86 | 5.24 |  |
|  | **Pyrimidine metabolism** |  | 1.56 | 1.52 |  |
|  |  | carbamoyl-phosphate synthase | 0 | 0 | [K01954](http://www.genome.jp/dbget-bin/www_bget?ko:K01954) |
|  |  | aspartate carbamoyltransferase | 0 | 0 | [K00608](http://www.genome.jp/dbget-bin/www_bget?ko:K00608) |
|  |  | dihydroorotase | 0.18 | 0.11 | [K01465](http://www.genome.jp/dbget-bin/www_bget?ko:K01465) |
|  |  | dihydroorotase dehydrogenase | 0 | 0 | [K00254](http://www.genome.jp/dbget-bin/www_bget?ko:K00254) |
|  |  | orotate phosphoribosyltransferase | 0.03 | 0.02 | [K00762](http://www.genome.jp/dbget-bin/www_bget?ko:K00762) |
|  |  | orotidine-5'-phosphate decarboxylase | 0.04 | 0.03 | [K01591](http://www.genome.jp/dbget-bin/www_bget?ko:K01591) |
| Metabolism, Xenobiotics Biodegradation and Metabolism |  |  | 0.28 | 0.44 |  |
| Unclassified, Cellular Processes and Signaling |  |  | 4.80 | 5.51 |  |
| Unclassified, Genetic Information Processing |  |  | 4.14 | 3.75 |  |
| Unclassified, Metabolism |  |  | 5.70 | 5.70 |  |
| Unclassified, Poorly Characterized |  |  | 4.46 | 4.74 |  |

Table S5: Comparison of main pathways in metabolomes and metagenomes of green and red snow.

|  | Metabolomics [%] | | Metagenomics [%] | |
| --- | --- | --- | --- | --- |
|  | Green snow | Red snow | Green snow | Red snow |
| purine metabolism | 30.0 | 62.8 | 5.9 | 5.2 |
| pyrimidine metabolism | 1.5 | 3.6 | 1.6 | 1.5 |
| tryptophan metabolism | 12.9 | 3.0 | 0.3 | 0.2 |
| mannitol cycle | 0.4 | 0.8 | 0.8 | 1.0 |
| sphingolipid metabolism | 4.5 | 12.9 | 0.0 | 0.2 |

Table S6: Number of raw and processed sequences for amplicons and metagenomes.

|  | Green snow | | Red snow | |
| --- | --- | --- | --- | --- |
|  | raw sequences | after QC | raw sequences | after QC |
| Eukaryotes | 7552 | 4334 | 8292 | 4595 |
| assigned to algae |  | 3685 |  | 3131 |
| Bacteria | 7568 | 3100 | 15665 | 4011 |
| Archaea | 162714 | 154711 | 65697 | 57661 |
| Metagenomes | 504879 | 343339 | 261005 | 184871 |
